# Paleorecords Reveal Biological Mechanisms Crucial for Reliable Species Range Shift Projections Amid Rapid Climate Change

**DOI:** 10.1111/ele.70080

**Published:** 2025-02-18

**Authors:** Victor Van der Meersch, Edward Armstrong, Florent Mouillot, Anne Duputié, Hendrik Davi, Frédérik Saltré, Isabelle Chuine

**Affiliations:** ^1^ CEFE, Univ Montpellier, CNRS, EPHE, IRD Montpellier France; ^2^ Department of Geosciences and Geography University of Helsinki Helsinki Finland; ^3^ UMR 8198‐EEP‐Evo‐Eco‐Paleo Université de Lille, CNRS Lille France; ^4^ URFM, INRAE Avignon France; ^5^ Biogeography Ecology and Modelling, School of Life Sciences University Technology Sydney Sydney New South Wales Australia; ^6^ Australian Museum Research Institute Australian Museum Sydney New South Wales Australia; ^7^ ARC Centre of Excellence for Indigenous and Environmental Histories and Futures James Cook University Cairns Queensland Australia

**Keywords:** climate change, ecological modelling, hindcasting, model transferability, species range shift

## Abstract

The recent acceleration of global climate warming has created an urgent need for reliable projections of species distributions, widely used by natural resource managers. Such projections have been mainly produced by species distribution models with little information on their performances in novel climates. Here, we hindcast the range shifts of forest tree species across Europe over the last 12,000 years to compare the reliability of three different types of models. We show that in the most climatically dissimilar conditions, process‐explicit models (PEMs) tend to outperform correlative species distribution models (CSDMs), and that PEM projections are likely to be more reliable than those made with CSDMs by the end of the 21st century. These results demonstrate for the first time the often promoted albeit so far untested idea that explicit description of mechanisms confers model robustness, and highlight a new avenue to increase model projection reliability in the future.

## Graphical abstract

Climate change has created an urgent need for reliable projections of species distributions. By hindcasting forest tree range shifts across Europe over the last 12,000 years, we show that process‐explicit models tend to outperform correlative models in novel climates, suggesting that mechanistic understanding and representation is critical to provide more reliable projections under future climate scenarios.

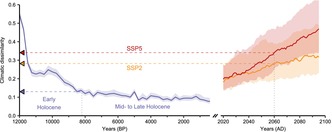

## 1 Introduction

Credible model projections are critical for natural resource managers, decision makers and stakeholders to make informed decisions. To meet the demand for reliable projections of ecosystems and biodiversity dynamics, comprehensive assessments of ecological model performances must be a priority (Dawson et al. 2011; Mouquet et al. 2015; Pacifici et al. 2015).

One approach to evaluate model reliability is to compare their predictions to observations from previous time periods, that is, hindcasting. Hindcasting can inform whether models capture, implicitly or explicitly, the essential processes required to provide reliable projections in conditions significantly different from the present. By looking far into the past, paleo‐archives have proven to offer a unique opportunity to both understand long‐term climate and biodiversity dynamics (Bartlein et al. 2011; Fordham et al. 2020) and test model robustness and transferability (Braconnot et al. 2012; Maguire et al. 2015)—that is, model capacity to maintain its performance in changing conditions (Uribe‐Rivera et al. 2023).

Yet, models' predictions of past species distribution and biosphere functioning rarely align with paleoclimate reconstructions and fossil records (Veloz et al. 2012; Pearman et al. 2008; Roberts and Hamann 2012; Foley et al. 2013; Maguire et al. 2016). Interpreting model projections in climatic conditions that differ significantly from the present, such as future no‐analogue climatic conditions (Williams et al. 2007), remains challenging. Therefore, the guarantee that ecological model forecasts for the 21st century will be reliable is limited (Fitzpatrick et al. 2018).

**FIGURE 1:**
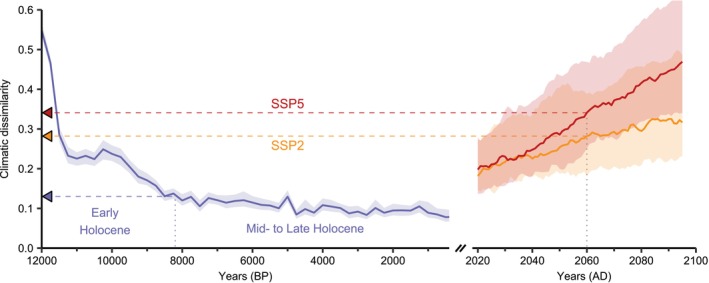
Evolution of climatic dissimilarity during the Holocene (12k–500 yr BP) and the 21st century (2020–2100), relative to 1901–2000. Climatic dissimilarity is computed as 1‐Sørensen similarity between bootstrapped climatic hypervolumes. Lines represent median dissimilarity, shaded areas represent 90% confidence intervals. Blue corresponds to paleoclimate based on HadCM3B model (30‐year period each 250 years). Yellow and red correspond to future climatic conditions according to SSP2‐4.5 and SSP5‐8.5 scenarios respectively, predicted by 34 global climate models of NEX‐GDDP‐CMIP6 (10‐year moving window). The blue triangle on *y*‐axis indicates the level of climatic dissimilarity at 8200 years BP, the limit between the early and mid‐ to late Holocene. Yellow and red triangles indicate the expected level of climatic dissimilarity in 2060 for SSP2‐4.5 and SSP5‐8.5 scenarios. Note that the *x*‐axis scale is different between past and future panels.

While exact matches to expected 21st‐century climatic conditions do not exist in historical records (Burke et al. 2018), the dissimilarity between 20th and 21st century median climatic conditions (Section 2) falls within the range of dissimilarity encountered since the beginning of the Holocene (12 kyr Before Present [BP]; Figure 1). This period takes place after the Last Glacial Maximum (26.5–19 kyr BP; Clark et al. 2009) and began with an abrupt climate warming followed by a long, almost uninterrupted, period of climatic stability until recent anthropogenic warming (Figure S4). The fossil pollen data accumulated over these last millennia provides us with a unique extended timeframe to test the reliability of ecological models, in particular those designed to predict changes in species distribution.

Species distribution models are powerful tools to predict species geographical distribution as a function of environmental data (e.g., mean annual temperature and annual total precipitation). Most studies have focused on correlative species distribution models (CSDMs, or niche models), which infer statistical relationships between observations of species occurrences and environmental predictors (Dormann et al. 2012). Their high flexibility and low computational complexity make them the most widely used tool for deciding on species conservation plans and policy regimes (e.g., Hanewinkel et al. 2013). However, under novel climatic conditions, new unobserved portions of a species' climatic niche may appear, which are not captured by these correlative (or phenomenological) approaches. For example, when tested under distant past climates, the predictive performance of CSDMs significantly decreased (Maguire et al. 2016), questioning their ability to provide reliable projections in the future (Fitzpatrick et al. 2018). However, these discrepancies might be partly due to dispersal constraints which can create a disequilibrium between species distribution and climate (Svenning and Skov 2004).

Alternative approaches to CSDMs are process‐explicit models (PEMs, or process‐based models) that rely on explicit formulations of the mechanisms driving the distribution of a given species (e.g., physiological, ecological and/or demographic processes). They come from decades of experiments and observations, including extreme conditions in laboratory (Seehausen et al. 2017), and climate manipulations such as CO_2_ enrichment (Jiang et al. 2020) or rainfall exclusion (Gavinet et al. 2019). The reliability of PEMs depends on our level of understanding of how environmental conditions affect ecophysiological processes and the availability of large amount of observations to calibrate their many parameters (Evans et al. 2016). Because these models do not rely on statistical relationships between present‐day species occurrences (presence/absence) and environmental variables, but rather describe explicit causal relationships between biological processes and environmental variables, they are believed to provide more reliable predictions of species distribution changes under novel climatic conditions (Evans 2012; Singer et al. 2016). However, another possible reason why PEM projections might be more reliable than CSDM projections under novel climatic conditions could also come from their calibration methods. Unlike CSDMs that are calibrated using species presence/absence data, PEM parameters are either measured directly (e.g., specific leaf area, leaf frost hardiness), or inferred statistically when direct measurement is not an option, using data on specific functional traits measured in the field or in laboratory (e.g., parameters of bud dormancy break date models).

The assumption that PEMs could provide more reliable projections of future range shifts of species is widely accepted and taken for granted (Evans 2012; Connolly et al. 2017; Urban et al. 2016; Pilowsky et al. 2022) although it has never really been demonstrated. Furthermore, the reasons behind this assumption have not been clearly articulated. Qualitative models comparisons under future climatic conditions have shown that PEMs often make more conservative projections in future climates than CSDMs which predict larger changes (Morin and Thuiller 2009; Cheaib et al. 2012; Gritti et al. 2013) but they have not provided any confidence level in these results. Very few studies have actually gone beyond qualitative comparisons between CSDMs and PEMs and compared thoroughly their performance, for example using virtual species (Zurell et al. 2016), exotic species in native and newly colonised areas (Higgins et al. 2020), or in the recent past (Fordham et al. 2018). While PEMs have shown their usefulness for paleoecological studies (Saltré et al. 2013; Ruosch et al. 2016; Schwörer et al. 2014), the extent to which they can provide more reliable predictions than CSDMs under different climatic conditions from the historical period remains unknown (Uribe‐Rivera et al. 2023; Briscoe et al. 2019).

Here, we address this critical gap by using multiple CSDMs and PEMs to simulate paleodistributions of five emblematic tree species of Europe at a high temporal resolution since 12 kyr BP. We used daily paleoclimatic data at 0.25° spatial resolution, generated from HadCM3B‐M2.1 coupled general circulation model simulations, which includes both inter‐annual variability, and millennial scale variability for rapid Dansgaard–Oeschger events before 11 kyr BP (Armstrong et al. 2019). Species migration ability was also incorporated into the simulations to represent more comprehensively changes in species' realised distribution and not merely changes in their climatic niches to allow for a more accurate comparison with the paleorecords.

We first assessed which modelling approach best predicts past species distributions, and second whether model performance was related to their hypotheses (relationships describing explicit biological mechanisms or not) or to their calibration methods (calibrated on species occurrence data or not). To do so, we compared three types of models: CSDMs, PEMs (hereafter called expert PEMs) and fitted PEMs calibrated in the same way as CSDMs (inverse calibration using species occurrence data and a novel type of algorithm; Section 2 and Van der Meersch and Chuine 2023). The comparison between CSDMs/fitted PEMs and expert PEMs allowed us to determine whether the differences in model performance arise from their calibration methods, whereas the comparison between CSDMs and expert/fitted PEMs allowed us to determine whether the differences in model performance arise from the model hypotheses. We evaluated model performance over the past 12,000 years, covering levels of climate dissimilarity comparable to those expected by the end of this century (Figure 1).

## 2 Methods

### 2.1 Correlative and Process‐Explicit Species Distribution Models

We used PHENOFIT and CASTANEA, two process‐explicit models which differ by their underlying hypothesis and complexity. PHENOFIT simulates the fitness of an average adult tree (Chuine and Beaubien 2001). It estimates fitness components (survival and reproductive success) by simulating the precise phenology (dates of leaf unfolding, flowering, fruit maturation and leaf senescence) and damages caused by abiotic stress (frost and drought). The effects of these stresses depend on their timing relative to the developmental stages of the plant's organs. It has been validated for several North American and European species (Morin et al. 2007; Saltré et al. 2013; Duputié et al. 2015; Gauzere et al. 2020). The model has ~30 parameters. CASTANEA simulates carbon and water cycles of an average adult tree by simulating many processes such as photosynthesis, stomatal opening, maintenance and growth respiration, transpiration and carbon allocation (Dufrêne et al. 2005). It has been used to predict carbon and water budgets of several European species (Davi et al. 2006; Delpierre et al. 2012; Davi and Cailleret 2017). The model has ~80 parameters. Both models require daily meteorological variables and soil characteristics. Two versions of both models were employed. The parameters of the first version—called *expert*—were either directly measured, or found in the literature, or calibrated using observations and measurements of the processes modelled. This version explicitly incorporates expert knowledge—for example, to discard parameter values that would fall outside the expected range (according to experimental results) in case of similarly efficient parameter sets. The second version—called *fitted*—was entirely calibrated using species distribution data like correlative models (Van der Meersch and Chuine 2023). For the latter, we used the optimisation algorithm CMA‐ES (Hansen and Ostermeier 2001) as described in Van der Meersch and Chuine (2023), and retained the best calibrations in terms of AUC (see Supplementary Methods for further details).

We selected correlative models based on the thorough model comparison made by Valavi et al. (2022). Among the most performant models, we selected five well‐established models: GLM with lasso regularisation, GAM, BRT, MaxEnt and down‐sampled Random Forest (see Supplementary Methods for further details). Some of these models are known to struggle when applied to extrapolation domains, but are nevertheless widely used by ecologists to provide projections of species distribution change in future climatic conditions. We selected four uncorrelated climate predictors based on their relevance to key ecological processes that are known to impact the distribution of forest trees and are represented in process‐explicit models. The minimum temperature of the coldest month was chosen to represent frost tolerance, which is crucial for tree survival during winter. Total precipitation was chosen to reflect the availability of water which is essential for tree growth and survival. Growing degree days (> 5°C) between April and September was chosen to represent the thermal energy available for vegetation growth and fruit maturation. The water balance between June and July (precipitation minus evapotranspiration) provides an indication of the intensity of summer drought, which is crucial for tree survival during summer. In addition, we included two soil covariates, pH and water holding capacity, to account for soil properties that influence water availability and nutrient uptake. Note that selecting a subset of these models (e.g., only the high‐performance ones according to Valavi et al. 2022) does not alter the results presented below (Figure S14).

While by construction correlative models directly output species habitat suitability, we used fitness predicted by the model PHENOFIT and net primary production predicted by the model CASTANEA as a proxy of species habitat suitability as they have already been used to predict species presence in previous studies (Morin and Thuiller 2009; Cheaib et al. 2012; Saltré et al. 2013). CSDMs and inverse‐calibrated PEMs were calibrated for five species ( *Fagus sylvatica* L., *Abies alba* Mill., *Quercus robur* L., *Quercus petraea* [Matt.] Liebl. and *Quercus ilex* L.) using climate variables (1970–2000) extracted from ERA5‐Land hourly dataset (Muñoz‐Sabater et al. 2021), soil data from EU‐SoilHydroGrids (Tóth et al. 2017) and SoilGrids (Hengl et al. 2017) databases and species presence data from the dataset assembled in Van der Meersch and Chuine (2023), mostly based on EU‐Forest inventory data (Mauri et al. 2017; see Supplementary Methods and Figure S1 for further details). To calibrate the CSDMs, we additionally sampled 50,000 background points, which should properly represent the variation in the environmental conditions across the study area (Valavi et al. 2022). For each CSDM and each species, we run a fivefold environmental cross‐validation to estimate model performance in novel extrapolation conditions (Figure S12; Roberts et al. 2017). We then used all the available training data to calibrate the models for the hindcasting in order to favour final prediction quality (Roberts et al. 2017). We could not run the same cross‐validation method for fitted process‐explicit models because it would have been too computationally expensive.

Model simulations over the Holocene were run for 30‐year centred periods—the standard length recommended by the World Meteorological Organization—every 250 years, for the five above mentioned species. Model outputs were averaged over each 30‐year period. Note that soil conditions (needed both for correlative and process‐explicit models) were held constant throughout the simulations, and were bilinearly interpolated from closest coastal cells where data was missing (because of different land‐sea masks between present and past). Note also that for CASTANEA model, species‐specific thresholds of net primary production determining the presence or absence of the species were computed with the CO_2_ level at the beginning of the Holocene (~240 ppm).

### 2.2 Holocene Climate and Vegetation

We used the monthly paleoclimate simulation dataset generated with the HadCM3B‐M2.1 coupled general circulation model (Armstrong et al. 2019), starting from 18 kyr BP at 0.5° spatial resolution for Europe (Figure S4). We chose this dataset for several reasons. First, it includes both inter‐annual variability, and millennial scale variability for rapid Dansgaard–Oeschger events before 11 kyr BP. Second, it shows generally a good agreement with ice‐core datasets (Armstrong et al. 2019). Third, it provides all the necessary input variables necessary to run all the models selected. For this work, several variables were specifically produced: mean temperature, average minimum and maximum daily temperatures, precipitation, number of rainy days, cloudiness and wind speed. We further downscaled temperature and precipitation monthly data to 0.25° resolution, by applying an elevation correction of coarse‐scale variables towards the ICE‐6G‐C elevation level at high resolution (Peltier et al. 2015). We then generated daily data for temperatures, precipitation, cloud cover and wind speed from the monthly data with the weather generator GWGEN (Sommer and Kaplan 2017), for 30‐year centred periods every 250 years. We also simulated daily extra‐terrestrial solar radiation with the same orbital forcing conditions used in HadCM3B‐M2.1 (Armstrong et al. 2019) and then computed daily global radiation taking into account previously generated daily cloud‐cover data as implemented in LPJ‐LMfire global model (Pfeiffer et al. 2013). Finally, we computed daily potential evapotranspiration following the standard FAO Penman‐Monteith method (Allen et al. 1998). Note that for smaller plants and shrubs, such macroclimatic variables may overestimate species range shifts (Maclean and Early 2023).

Fossil pollen records were extracted from the LegacyPollen dataset (Herzschuh et al. 2022). This dataset is mainly based on the Neotoma database (Williams et al. 2018), and provides samples with standardised chronologies and age uncertainties. We removed sites that had marine depositional environments (Maguire et al. 2016), and only kept samples with more than 200 pollen grain counts and age uncertainty of less than 500 years. Pollen relative abundances were aggregated to consecutive 500‐year intervals. If multiple samples from the same site belonged to the same period, we averaged their pollen abundances, weighting by their age uncertainty and temporal distance from the centre of the period (Figure S2). Relative pollen abundances were converted to presence/absence using thresholds based on biome reconstructions (Williams et al. 1998): 1% for *Fagus* and *Abies* and 2.5% for *Quercus*. If several sites fell within the same grid cell (0.25°), we considered the species as present if there was at least one site where the species could be considered as present. Note that not all 0.25° grid cells within the study area are covered by pollen data (due to the high spatial and temporal variability of pollen records). As a result, model evaluations were conducted only for grid cells where species presence or absence data were available. *Fagus* pollen data were used to assess the presence of *Fagus sylvativa* L., sole species of the genus present in Europe. *Abies* pollen data were used to assess the presence of *A. alba* Mill., the most abundant and widespread fir species present in Europe. When possible, deciduous and evergreen *Quercus* pollen were distinguished based on Neotoma data. Some *Quercus* pollen remain undetermined beyond the generic level, either because discrimination between evergreen and deciduous oak pollen was impossible or because authors were not specific. They were assigned to two categories, based on the evergreen natural range as defined by Atlas Flora Europeae (Jalas and Suominen 1972–2005) and EuroVegMap (Bohn et al. 2003): pollen outside range were considered as deciduous only occurrences, whereas pollen inside range were considered as both evergreen and deciduous occurrences. Deciduous *Quercus* pollen data were used to assess the presence of *Quercus petraea* (Matt.) Liebl. and *Q. robur* L., the two most abundant and widespread deciduous oak species in Europe. Evergreen *Quercus* pollen data were used to assess the presence of *Quercus ilex* L., the most abundant and widespread evergreen oak species in Europe.

### 2.3 Tree Migration

Models used in this study predict species potential distribution based solely on climatic and soil conditions. To compare model predictions to pollen paleorecords, species migration needs to be simulated as well, as it can be the primary factor limiting species distribution before climatic conditions, especially when climatic conditions are changing rapidly as it was the case during the Dansgaard–Oeschger events (Svenning and Skov 2004; Saltré et al. 2013).

To implement migration in the simulations, we ran a cellular automaton (Engler et al. 2012) which has proven to be as accurate as more complex approaches (Zurell et al. 2016). We modified the initial version of this dispersal model in order to use both short‐ and long‐distance dispersal kernels (long distance events could occur with a probability of 0.01). We used species‐specific fat‐tailed kernels (Zani et al. 2022) at a 500 m resolution, and assumed that trees can disperse once a year (Figure S11a). SDM outputs were assigned to two classes using specific optimal thresholds maximising model performance (TSS) in the 1970–2000 period (Figure S9): (i) cells where the model output was under the specific threshold were assigned a zero suitability (species cannot survive) and (ii) cells where the model output was above the threshold, the suitability was rescaled between 0 and 1 (species can migrate), representing the probability of a cell to become colonised. We considered the deciduous *Quercus* suitability as the maximum suitability between *Q. robur* and *Q. petraea* . Migration simulations started from 12 kyr BP (or 11.75 kyr BP when a model simulates no presence at 12 kyr BP, Figure S10), and the suitability simulated by SDM was updated every 250 years (see Supplementary Methods and Figure S3 for further details). Starting at 11.75 kyr BP or 12 kyr BP does not change our results (Figure S16), and we could not start earlier (e.g., 15 kyr BP) as most models predict no presence at all around 12.5 kyr BP. Additionally, we ran migration simulations starting from 11.5, 11.25 and 11 kyr BP to investigate the effect of initial climatic dissimilarity on our results (Figure S16). We also checked that dispersal process stochasticity at 500 m resolution (Figure S11a) had no significant effect on the model's performance at the scale of Europe, by simulating deciduous *Quercus* migration 10 times for each of the nine models (Figure S11b). We could have referred to the initial starting points—predicted by the models at 12 or 11.75 kyr BP—as refugia. However, we chose to avoid using this term in the following, as it is commonly associated with finer microclimatic scales and the long‐term persistence of populations.

### 2.4 Models' Performance

We used the Sørensen's similarity index to measure the hindcast performance of the models, based on the confusion matrix. This discrimination measure has been shown to provide adequate estimations of model discrimination capacity, not biased by species prevalence or an inflated number of true negative predictions (Leroy et al. 2018). This feature is important when working with fossil pollen data, for which the number of species absence can be much higher than the number of species presence. Note that we obtained similar results when using TSS as the performance metric (Figure S15b). We compared the area potentially occupied (not taking migration into account) and occupied (taking migration into account) by the species to the presence/absence data extracted from the LegacyPollen dataset every 500‐year interval. Kruskal–Wallis tests followed by multiple pairwise post hoc Conover‐Iman tests (as implemented in the R package *conover.test*) were computed to assess stochastic dominance among model performance and transferability (Figure 3b–d).

To quantify the climatic differences between historical climate (1901–2000, based on the CRU TS v. 4.07 gridded dataset; Harris et al. 2020) and Holocene climate (hindcasting conditions), we computed the *climatic dissimilarity* as the Sørensen dissimilarity between climatic hypervolumes (a metric of overlap in multidimensional space). We first generated for each period (500‐year intervals from 12 kyr BP to 500 BP and 1901–2000) a set of 20 bootstrapped hypervolumes, using R package *hypervolume* (Blonder et al. 2018). Hypervolumes were computed with a Gaussian kernel density estimation method based upon the first three principal component axis from 3‐month means temperature and 3‐month sums of precipitation. We then computed overlap statistics (mean and standard deviation of Sørensen index) between the bootstrapped hypervolumes of each time points of the Holocene and the bootstrapped hypervolumes of the historical period (i.e., 20 × 20 overlaps). As a comparison, we also computed the climate novelty based on Mahalanobis distance (Figure S5; Burke et al. 2019).

We also computed these metrics under future conditions to compare the dissimilarity of future climate to that of the Holocene climate, both relative to 20th century climate. To assess future conditions, we used all the global climate models from NEX‐GDDP‐CMIP6 dataset (Thrasher et al. 2022)—except HadGEM3‐GC31‐MM, not available for SSP245—and 2 scenarios (SSP245 and SSP585). To make the comparison, both paleoclimate and future climate data were uniformised with the CRU dataset to maximise comparability between paleoclimate and future climates. The difference (for 3‐month temperature average) and the ratio (for 3‐month precipitation sum) between the observations (from 1901 to 2000) and simulations (1901–1950 for HadCM3B and 1951–2000 for CMIP6 projections) were calculated and applied to the whole modelled time period, assuming that the bias was constant.

Finally, we estimated the effects of past climate novelty (Sørensen's climatic dissimilarity) on model performance (Sørensen index) with a Bayesian ordered beta regression, considering the different types of models (correlative, fitted process‐explicit and expert process‐explicit), using the R package *ordbetareg* (Kubinec 2023) and RStan (Stan Development Team 2023). Compared to a standard beta regression model, this model allows for observations at the bounds (i.e., Sørensen index = 0 or = 1). We took into account the standard deviation of Sørensen's climatic dissimilarity (computed with sets of bootstrapped hypervolumes, see above) as a predictor measurement error.

## 3 Results

As observed in previous long‐term historical assessments, all models showed a decrease of their performance when moving further into the past, that is, into more different climatic conditions than historical conditions (Figure 3a). However PEMs showed smaller decrease in their predictive performance (slope of Beta regression, fitted PEMs: −6.07, 95% CI [−8.62, −3.55], expert PEMs: −4.44, 95% CI [−7.07, −1.77]) than CSDMs (−11.0, 95% CI [−13.2, −8.91]). PEMs also showed higher transferability in the most distant climatic conditions of the early Holocene than CSDMs (Figure 3d). PEMs, either expert or fitted, are thus less affected by the increase in climate dissimilarity than CSDMs. In the near past (Mid‐ to Late Holocene, < 8.2 kyr BP), CSDMs were not significantly better at predicting tree distribution than any PEMs (pairwise Conover‐Iman tests: vs. expert PEMs *t*‐statistic = −1.68/*p* < 0.128, vs. fitted PEMs *t*‐statistic = −1.55/*p* < 0.112; Figure 3b), despite their closer fit to current species distributions (Figure S12). In the distant past (early Holocene, > 8.2 kyr BP), CSDMs performed worse than both expert and fitted PEMs (pairwise Conover‐Iman tests: respectively *t*‐statistic = −4.80/*p* < 0.0001 and *t*‐statistic = −5.07/*p* < 0.0001; Figure 3b). The maximum climatic dissimilarity during this period corresponds to the climatic dissimilarity expected as soon as 2060 according to the scenario SSP245 (Figure 3a).

**FIGURE 2:**
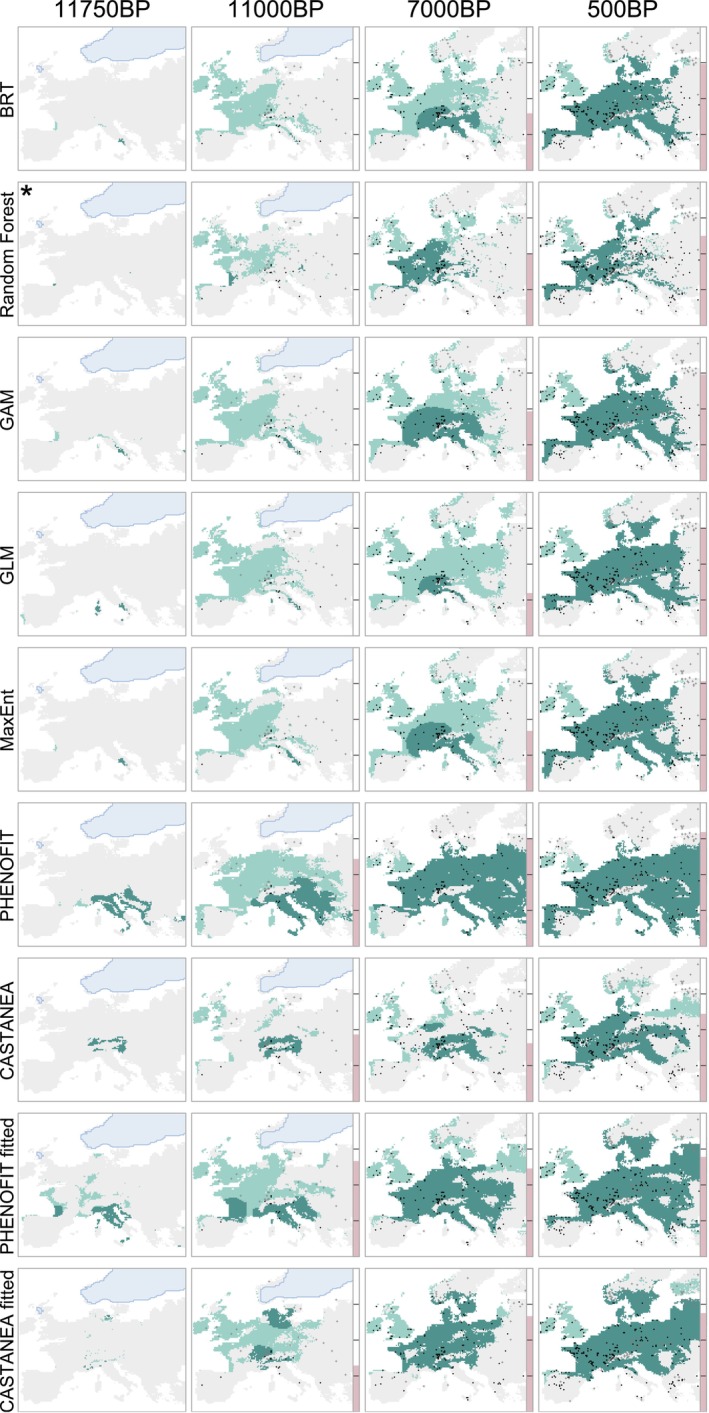
Example of paleosimulations obtained with the nine models used in this study for deciduous oaks. The five first rows correspond to the five correlative models (boosted regression tree, down‐sampled random forest, generalised additive model, generalised linear model with lasso regularisation and MaxEnt). The four last rows correspond to two different versions (expert calibration and inverse calibration using occurrence data) of two process‐explicit models (PHENOFIT and CASTANEA). Light green area is the modelled suitable area, dark green area is the colonised area (after migration). Light blue represents the ice sheet extent. Black dots are deciduous oak presences (based on fossil pollen), grey crosses are absences. The vertical pink gauge represents model predictive performance (Sørensen index, [0,1], ticks every 0.25). The model for which migration started at 11.75 kyr BP rather than 12 kyr BP is marked with an asterisk. ‘BP’ stands for ‘before present’ (1950). See Figures S7–S9 in the Supporting Information for the same maps for beech, fir and evergreen oak.

Differences between PEMs and CSDMs projections are closely related to their ability to predict species recolonisation dynamics in the Early Holocene (~11.5–8.5 kyr BP, Figure 2), which in turn depends on the initial starting points predicted by the models—and therefore on the level of climatic dissimilarity during this initial period (12–11.75 kyr BP; Figure S10). Higher performance trends from these starting points could indicate that projections of PEMs—fitted and expert—were more accurate than those of CSDMs at this time period, although they could not be tested against fossil records (Figure 2). This period corresponds to a global deglaciation which lasted for a few centuries and occurred after the cooling of the Younger Dryas interval (~13–11.75 kyr BP; Figure S4). This rapid warming episode explains the strong decrease of climate dissimilarity relative to present between 12 kyr BP and 11.5 kyr BP (Figure 1). If we had not considered the 12–11.75 kyr BP period of high climatic dissimilarity (i.e., by simulating migration from 11.5 kyr BP), we would have missed the opportunity to take into account model projections within the same dissimilarity level to what we expect between 2050 and 2100 (Figure 1). When model projections with migration starting from 11.5 kyr BP or later are compared—that is, when climate dissimilarity is below 0.3 and thus more similar to present—CSDMs and PEMs' abilities to predict fossil pollen occurrence are similar (Figure S16).

**FIGURE 3:**
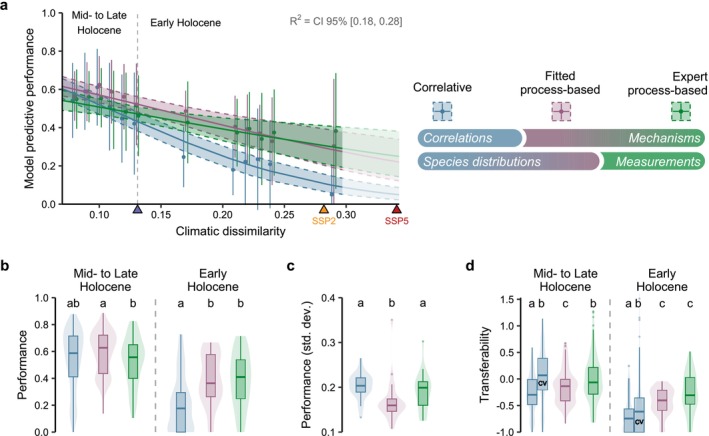
Performance of correlative models, fitted process‐explicit models (inverse calibration using occurrence data) and expert process‐explicit models (classical calibration) against Holocene paleoecogical evidence (fossil pollen) for 4 tree genera (*Abies*, *Fagus*, *Quercus* deciduous and *Quercus* evergreen). (a) Bayesian beta regression of model predictive performance (Sørensen index) against climatic dissimilarity relative to 1901–2000 (1‐Sørensen similarity between climatic hypervolumes). Shaded areas represent 2.5% and 97.5% quantiles of the posterior predictive distribution. Points represent the average model performance (and lines the standard deviation) grouped by similar level of climatic dissimilarity. Blue triangle on *x*‐axis indicates the limit between early Holocene (> 8.2 kyr BP) and mid‐ to late Holocene (< 8.2 kyr BP). Yellow and red triangles indicate the expected level of climatic dissimilarity in 2060 for SSP245 and SSP585 scenarios. See Figure S15a in the Supporting Information for the evolution of model performance displayed along a temporal *x*‐axis. Legend on the upper right: Top row represents drivers of modelled distributions (correlations/mechanisms), bottom row represents calibration method (species distributions/measurements). Panels (b) and (c) show the difference in performance (Sørensen index) and variability in performance (standard deviation of Sørensen index) across models. Panel (d) shows the transferability of the models (relative change in model performance between Holocene periods and 1970–2000 period). A negative transferability means that model performance is lower in Holocene periods than in the 1970–2000 period. CSDM predictive errors in the 1970–2000 period was assessed by two different methods: (i) against the same data used for calibration (leading to an overestimation of 1970–2000 model performance—but more comparable to fitted PEM estimates), (ii) using an environmental block cross‐validation, noted as ‘CV’ (leading to a better estimation of true model errors in the 1970–2000 period and thus a higher transferability—but less comparable to fitted PEMs for which cross‐validation would have been too computationally expensive). The grouping letters represent the multiple comparisons with pairwise Conover‐Iman tests.

Our results also revealed that inverse calibration improved process‐explicit projections (fitted PEMs) in recent past without altering significantly PEM long‐term transferability (Figure 3). In Mid‐ to Late Holocene, when climate conditions were not drastically different from present, performances of fitted PEMs was higher than those of expert PEMs (*t*‐statistic = 2.70/*p* = 0.020). In most distant climatic conditions of Early Holocene, their performances were similar (*t*‐statistic = 0.220/*p* = 0.757; Figure 3b).

Models performances were not stable across species, and exhibited both similarities and differences across time (Figure S13). More specifically, models exhibited the same overall performance decrease against *Fagus* pollen records, whereas CSDM performance decline was substantially faster than expert and fitted PEMs for deciduous *Quercus*. All models show low predictive power regarding evergreen *Quercus* distribution even in the late Holocene compared to other species, especially CSDMs which failed to predict its presence along the Atlantic coast (Figure S6). Fitted PEMs, however, showed the lowest variability of performance across species (Figure 3c).

## 4 Discussion

Our results suggest that the transferability and robustness of models are more strongly influenced by the processes explicitly represented in the models than by their method of calibration. PEMs show a better performance than CSDMs in the most climatically dissimilar conditions tested in this study (Figure 3, Figure S16), even when calibrated using the same method as CSDMs (i.e., fitted PEMs). Therefore, beyond enabling a more detailed mechanistic understanding of the effects of environmental conditions on species survival, growth, and reproduction, biological processes represented in PEMs are also critical to ensure higher model robustness in novel climates. This important new finding advocates for a wider use of PEMs to predict biodiversity and ecosystems distributions in the future and opens a new avenue to reach this goal by using inverse modelling approaches to calibrate them.

Simulating migration allowed us to take into account the differences between the models under the most challenging conditions—that is, when the climate dissimilarity was at its greatest (12–11.75 kyr BP)—closely approximating what is projected for the end of the 21st century. This corresponds approximately to a level of climatic dissimilarity around 0.45–0.55 (Figure 1, Figure S16). Since the migration model is identical across all simulations, differences of performance between models across the Holocene very much depended on their ability to predict the potential distribution of the species during the Early Holocene (Figures S10 and S16). For example, some models were not able to predict evergreen *Quercus* occurrence in Southern Spain, thus missed an important migration route and failed predicting their presence in vast areas in the Late Holocene (Figure S9). As PEMs, either fitted or expert, describe the response of ecophysiological processes to a wide range of environmental conditions, they can provide a better estimate of the environmental conditions in which species could have survived 12,000 years ago, under climates much more dissimilar to present conditions. Model performances were indeed similar when starting simulations from 11.5 kyr BP or later—that is, when limiting the analysis to a climatic dissimilarity level below 0.3 (Figure S16). A potential limitation of our approach though is that we cannot account for very rare and really long‐distance dispersion events, as well as the influence of humans. For example, all models failed to predict deciduous *Quercus* in the British Isles before the early Holocene sea‐level rise and the opening of the Strait of Dover (Figure 2; Smith et al. 2011), even though the land‐sea mask changed throughout the simulations. It remains unclear whether this failure is due to the migration models' misrepresentation of very long‐distance dispersion events of seeds (e.g., by humans or jays, across major dispersal barriers), the result of historical contingency (possibly due to a single unlikely event), or a consistent misprediction by both CSDMs and PEMs of more northern initial occurrences (Figure S10).

The recent efforts to gather fossil pollen data and make them openly available (Williams et al. 2018) allow us to objectively assess model performance under climate conditions vastly different from those used for their calibration. From 11.5 kyr BP onwards, climate dissimilarity varies between 0.29 and 0.08, a level equivalent to what we might experience in the second quarter of the 21st century (Figure 1). The consistency of model projections with past observations does not demonstrate that model projections will be valid in the future (Oreskes et al. 1994), but making such comparisons allows to make a critical step towards enhancing our understanding of model transferability. As more and more pollen data becomes available, we could cover a wider range of conditions, notably prior 11.5 kyr BP. Our simulations nevertheless started at 12 kyr BP, when climatic dissimilarity was at its highest, and transitioned rapidly to a climate more analogous to historical state. The uncertainties on the initial conditions had thus a significant influence on the simulation outcomes. In the future, on the contrary, uncertainties on the initial conditions will be much lower as models will start from the known distributions of species, and uncertainties will increase as simulations proceed towards increasingly dissimilar climatic conditions, especially as these conditions will extend beyond the range experienced in the past (Figure S6). This makes it difficult to draw direct parallels between past and future model performance, but our findings suggest that PEMs may potentially be more reliable under future climatic conditions. By moving beyond ‘*what correlates with what*’ to ‘*why and how things happen*’, PEMs may provide a more robust framework—grounded in mechanistic principles—for understanding and predicting species range shifts.

While quantifying the uncertainty in model projections remains challenging, our results pave the way for drastic improvement in model evaluation. The discrepancies between model performances we observed highlight the importance of considering various modelling methods to capture the full range of uncertainties associated with future projections. It implies that we should not rely solely on the model's own prediction dispersion to estimate projection uncertainties, nor on very similar modelling approaches, especially when climate dissimilarity sharply increases. The rate of anthropogenic climate change and the increased probability of occurrence of novel climates (Figure 1; Williams et al. 2007) are challenging the reliability of both CSDMs and PEMs especially as they are intended to be used in more complex models such as biosphere‐atmosphere models and used by natural resource managers and policy makers to guide management plans and policies. Acknowledging these uncertainties is as important as making the forecasts themselves (Beale and Lennon 2012) and contributes to the public trust in scientists (Berkhout 2010). Moreover, models will have to consider that tree colonisation dynamics will likely be very different in the future because it will not only occur from a few locations but from wider continuous ranges, and direct anthropogenic factors, such as land‐use, sylvicultural practices and assisted species migration, will also shape the composition of forests (Aitken and Bemmels 2016; Guo et al. 2018; Ivory et al. 2019).

In this study, we focused on forest tree species for which we have a deep understanding of their functioning and a wealth of detailed measurements. Without this, it would not have been possible to develop process‐explicit equations or to parameterise expert PEMs. We thus believe that CSDMs remain highly valuable due to their simplicity and reliance on occurrence and environmental data that are more widely accessible. Notably, our results suggest that CSDMs perform relatively well under moderate levels of climatic dissimilarity and moderate rate of climate change, making them a reliable option in such contexts (Figure S16). While PEMs may provide improved predictions in rapidly changing environments—an essential feature for forest managers—the broad applicability of CSDMs still makes them a practical tool, especially in large‐scale biodiversity studies involving hundreds of species. Furthermore, there is potential to improve CSDMs by integrating more precise physiological data (e.g., Wagner et al. 2023), which could enhance their predictive power—yet there is currently no consensus on the robustness of these new approaches (Chevalier et al. 2024).

Fitted PEMs bring together the strengths from both CSDMs and expert PEMs approaches by describing causal relationships between environmental conditions and species performance (i.e., from process‐explicit approaches) and precise estimates of parameter values (from correlative approaches). The differences between expert and fitted PEMs in the mid‐ to late Holocene pinpoint some issues in expert parameterisation that requires to combine various methods to cope with both the scarcity of data for each ecophysiological process modelled and sometimes non‐measurable parameters (e.g., De Cáceres et al. 2023). Some parameters in these relations can be measured directly, and exhibit little variability across a species range (e.g., water potential leading to 50% of vessels embolism). However, the measurement of parameters in controlled conditions does not necessarily guarantee their external validity *in natura* (Asse et al. 2020) where numerous factors, not represented in laboratory conditions, can also affect the process modelled (but see Satake et al. 2013). Other parameters are in addition either highly variable because of local adaptation over long period, difficult‐to‐measure or so far unmeasurable (e.g., bud dormancy). Therefore, expert PEMs can suffer from uncertainties entailed in the measurements of some of their parameters, and from spurious data specific to few locations which do not represent sufficiently well all the conditions the species can experience all over its range. For these reasons, inverse calibration presents a valuable opportunity to estimate the values of PEM parameters especially difficult to estimate otherwise (Evans et al. 2016; Hartig et al. 2014). However, inverse calibration does not guarantee the correct estimation of parameter values and needs to be used critically and with caution (Van der Meersch and Chuine 2023).

Our unique multi‐model comparison across the Holocene demonstrates that our understanding of biological mechanisms embedded into process‐explicit models represent a real advantage over the empirical relationships used in CSDMs to increase projections reliability for the coming decades. However, data availability limits our ability to parameterise these models, and could explain the difficulty to use them more widely for global impact studies. Fitted PEMs may overcome this problem by using more data at a larger geographical scale, while keeping the predictive strength of causal relationships. Given ongoing improvements in computational methods and the availability of new global‐scale measurements (e.g., forest structure and growth with remote sensing and LiDAR data), extensive calibration and more widespread application of process‐explicit models seems now possible as well as an increase in model projections reliability.

## Author Contributions

**Victor Van der Meersch:** conceptualization, methodology, investigation, analysis, writing – original draft, writing – review and editing. **Edward Armstrong:** resources, writing – review and editing. **Florent Mouillot:** methodology, writing – review and editing. **Anne Duputié:** writing – review and editing. **Hendrik Davi:** methodology, writing – review and editing. **Frédérik Saltré:** conceptualization, writing – review and editing. **Isabelle Chuine:** conceptualization, methodology, writing – review and editing, supervision.

### Peer Review

The peer review history for this article is available at https://www.webofscience.com/api/gateway/wos/peer‐review/10.1111/ele.70080.

## Supporting information


Data S1.

## Data Availability

Simulation outputs, together with the code to reproduce the analysis and figures in this study, are available at https://doi.org/10.5281/zenodo.14681380, as well as on GitHub at https://github.com/vvandermeersch/past_robustness. GWGEN code is available at https://github.com/ARVE‐Research/gwgen_f90. Pollen records are available at https://doi.pangaea.de/10.1594/PANGAEA.929773.
