# Supplementary material for: Paleorecords Reveal Biological Mechanisms Crucial for Reliable Species Range Shift Projections Amid Rapid Climate Change
Source: Ecol Lett. 2025 Feb 18;28(2):e70080. doi: 10.1111/ele.70080 (PMC11836547; doi:10.1111/ele.70080)
Supplement: Supplementary file 1 — Data S1. [file ELE-28-0-s001.pdf]

Paleorecords reveal biological mechanisms crucial for reliable species range shift projections amid rapid climate change

Supplementary information

Victor Van der Meersch[1*], Edward Armstrong[2], Florent Mouillot[1], Anne Duputié[3], Hendrik Davi[4], Frédérik Saltré[5], Isabelle Chuine[1]

[1]CEFE, Université de Montpellier, CNRS, EPHE, IRD, Montpellier, France.
[2]Dept. of Geosciences and Geography, University of Helsinki, Helsinki, Finland.
[3]UMR 8198-EEP-Evo-Eco-Paleo, Université de Lille, CNRS, Lille, France.
[4]URFM, INRAE, Avignon, France.
[5]Global Ecology, College of Science and Engineering, Flinders University, Adelaide, Australia.

*Corresponding author(s). E-mail(s): victor.vandermeersch@cefe.cnrs.fr;

1 Supplementary Methods

1.1 Occurrence data used for the calibration of CSDMs and fitted PEMs

The occurrence data used in this study primarily come from the EU-Forest dataset [1]. This dataset is based on inventory and monitoring programs conducted across most European countries. Since it focuses only on forest ecosystems, we supplemented it with presence records from the Global Biodiversity Information Facility [2], while removing observations outside natural species ranges defined by the Atlas Florae Europaeae [3] and EuroVegMap [4]. This allowed us to include occurrences of isolated native trees outside forests, while excluding records from arboreta or gardens where species might have been planted as exotics. For holm oak, we also incorporated records from the Mediterranean Basin via the WOODIV database [5], excluding EU-Forest and GBIF records already gathered. We upscaled all species records to the ERA5-Land resolution (0.1°), considering a species present in a cell if at least one record was found. This workflow is summarized in Figure S1 below:

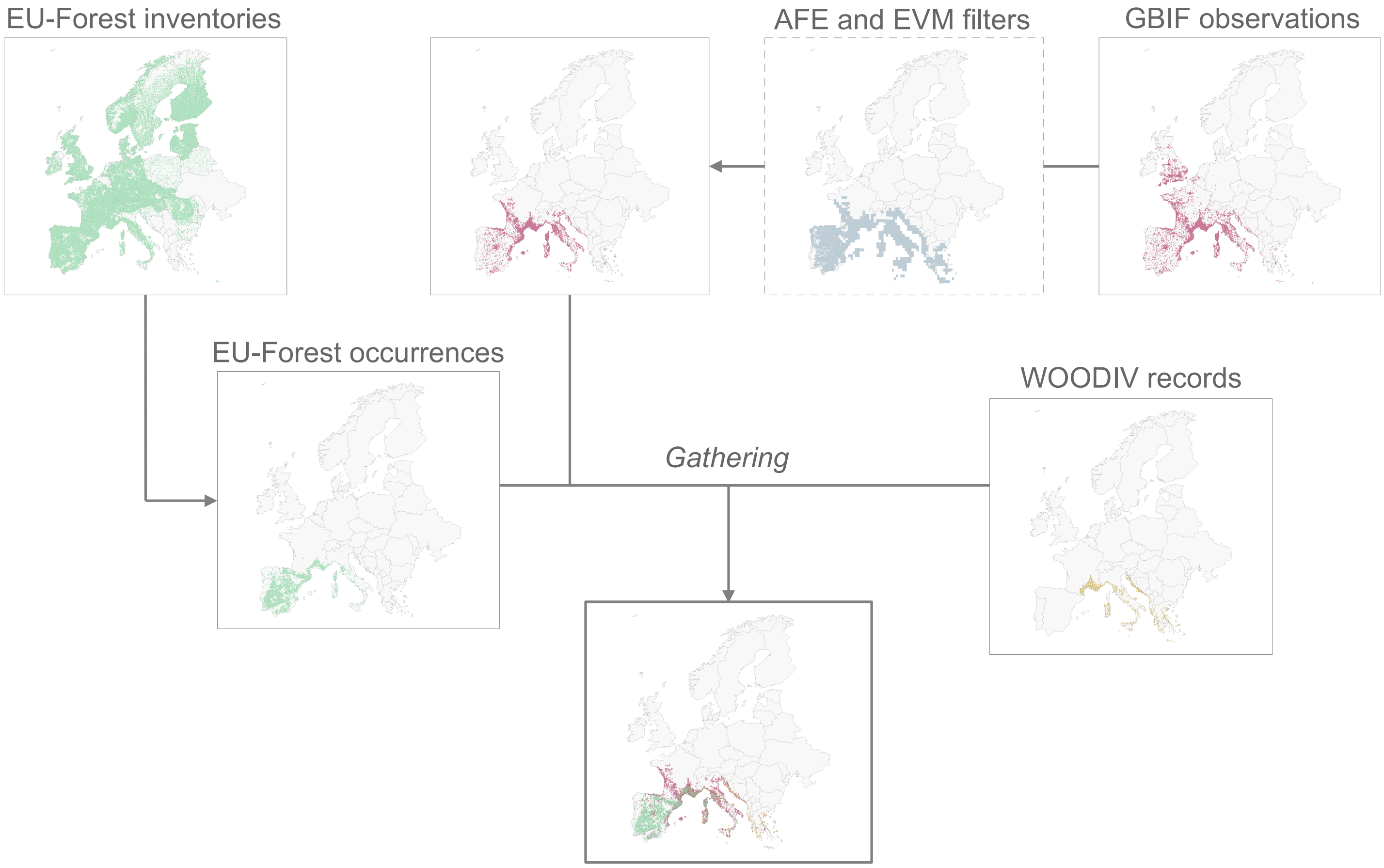

Fig. S1. Occurrence processing framework. Example with *Quercus ilex*.

1.2 Calibration of CSDMs

We selected five correlative models: GLM, GAM, BRT, MaxEnt and Random Forest. We applied the tuning recommendations of [6]:
- for GAM, GLM and BRT , background points were down-weighted so that their total weight was equal to the total weight of the presence points
- for GLM, we allowed both linear and quadratic terms and applied a lasso regularization which penalizes some coefficients to reduce their values until they eventually become zero, in order to avoid overfitting issues and improve model transferability. Following [6], the model was tuned with the internal cross-validation feature of *cv.glmnet* function in order to choose the parameter λ with the minimum deviance
- for MaxEnt, we kept the default tuning, as the tuned MaxEnt showed no statistically significant difference in performance in [6]
- for BRT, the model was tuned using the forward stepwise cross-validation approach implemend in the R package *dismo*, in order to estimate the optimal number of trees
- for Random Forest, each tree was fitted with a bootstrap sample of presences and the same number of background points, to avoid issues related to excessive Random Forest sensitivity to low ratios of presence and background data

1.3 Calibration of fitted PEMs

Fitted PEMs were calibrated using the procedure described in [7]. First, we selected subsets of 2000 points (1000 presences and 1000 absences) to minimize computational cost of inverse calibration. We employed a stratified random sampling to ensure that the environmental preferences of all species were proportionally represented.
We calibrated PHENOFIT and CASTANEA using the covariance matrix adaptation evolution strategy (CMA-ES), which is a robust algorithm for complex optimization problems [8]. It is inspired by Darwin's theory of evolution to find the most fit parameter sets. The objective function for the calibration was the area under the receiver operating characteristic curve (AUC), to maximize model discriminating capacity. We ran CMA-ES calibrations on two multicore clusters, GenOuest (genouest.org) and TGCC from CEA (hpc.cea.fr).
For PHENOFIT, we calibrated each species parameter set 10 times, using five repetitions on two random subsets of presences and pseudo-absences. Due to the significantly higher computing time required for CASTANEA, we performed only two calibrations for each species, each on a different random subset. For each species and each model, we kept the best calibration in terms of AUC.

1.4 Calibration of expert PEMs

On the contrary, the expert calibration does not involve species occurrence data at any point.
PHENOFIT (*expert version*) has been calibrated for several European tree species, and validated by comparing their historical and Holocene distribution to the modelled fitness [9–11]. Some parameters were directly measured or found in the literature, e.g. the frost hardiness parameters. Phenology-related parameters were inferred using phenological data across Europe – provided through the TEMPO data portal (data.pheno.fr) and the PEP725 database (pep725.eu).
The (*expert*) calibration of CASTANEA is even more complex due to its higher number of parameters. Most parameters came from previous works or were directly measured. Few parameters are prescribed based on expert knowledge as no data to estimate them exist. The complete parameterization of the model is given in the Table 1 in [12].

1.5 Pollen data used for SDM performance evaluation

As explained in the manuscript main text, when multiple samples from the same site belonged to the same period, their pollen abundances were averaged – weighting by their age uncertainty and temporal distance from the center of the period. It means that the closer a pollen record was from the center of the period considered and the smaller was its age uncertainty, the more its weight was important. This workflow is summarized in Figure S2 below:

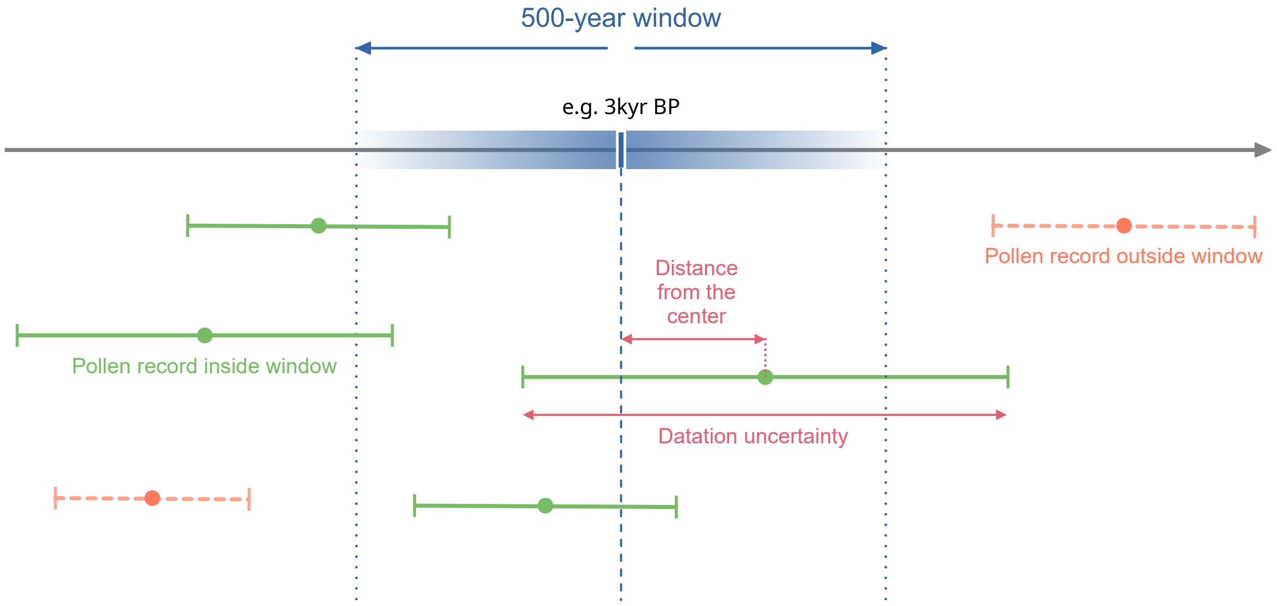

Fig. S2. Pollen processing framework. This illustrative example show a case when multiple pollen counts belonged to same 500-year window in the same site.

1.6 Simulations and model evaluation

SDMs are run for 30-year periods every 250 years. Then, starting from the suitable area predicted in 12kyr BP (or 11.75kyr BP), a migration cellular automation is run for 12000 years (or 11750 years) for each SDM. We started the migration simulations from 12 kyr BP (or 11.75 kyr BP when a model simulates no presence at 12 kyr BP) to simulate species dispersal from the coldest conditions. Unfortunately, we could not start earlier (e.g., 15 kyr BP), as most models predict no presence at all around 12.5 kyr BP—likely due to the coarse spatial resolution of our study. During the migration process, the suitable area is updated every 250 years. Finally, each SDM+migration output is tested against fossil pollen records for each genus, aggregated to 500-year intervals (see above). This workflow is summarized in Figure S3 below:

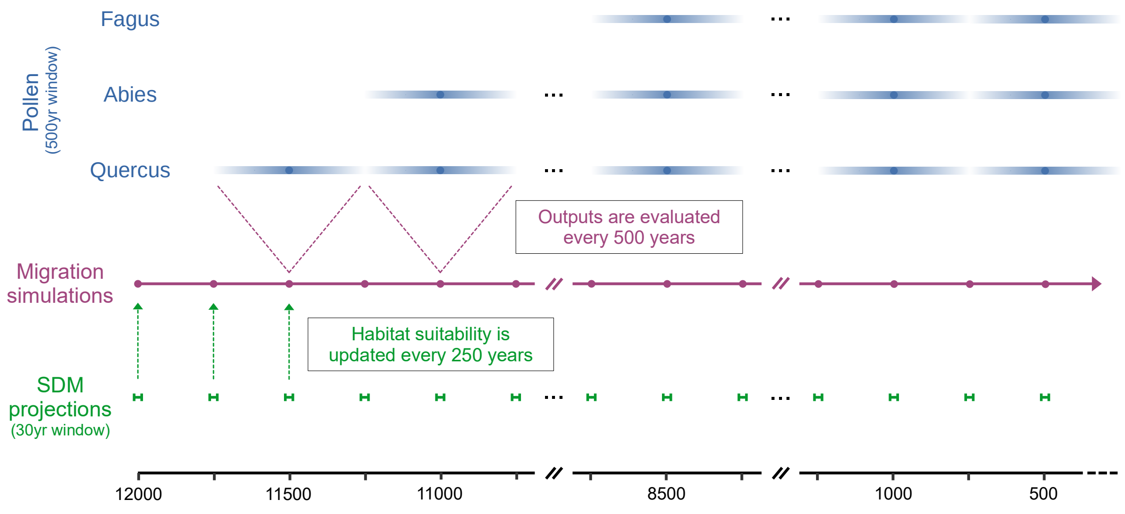

Fig. S3. Model simulation and evaluation framework.

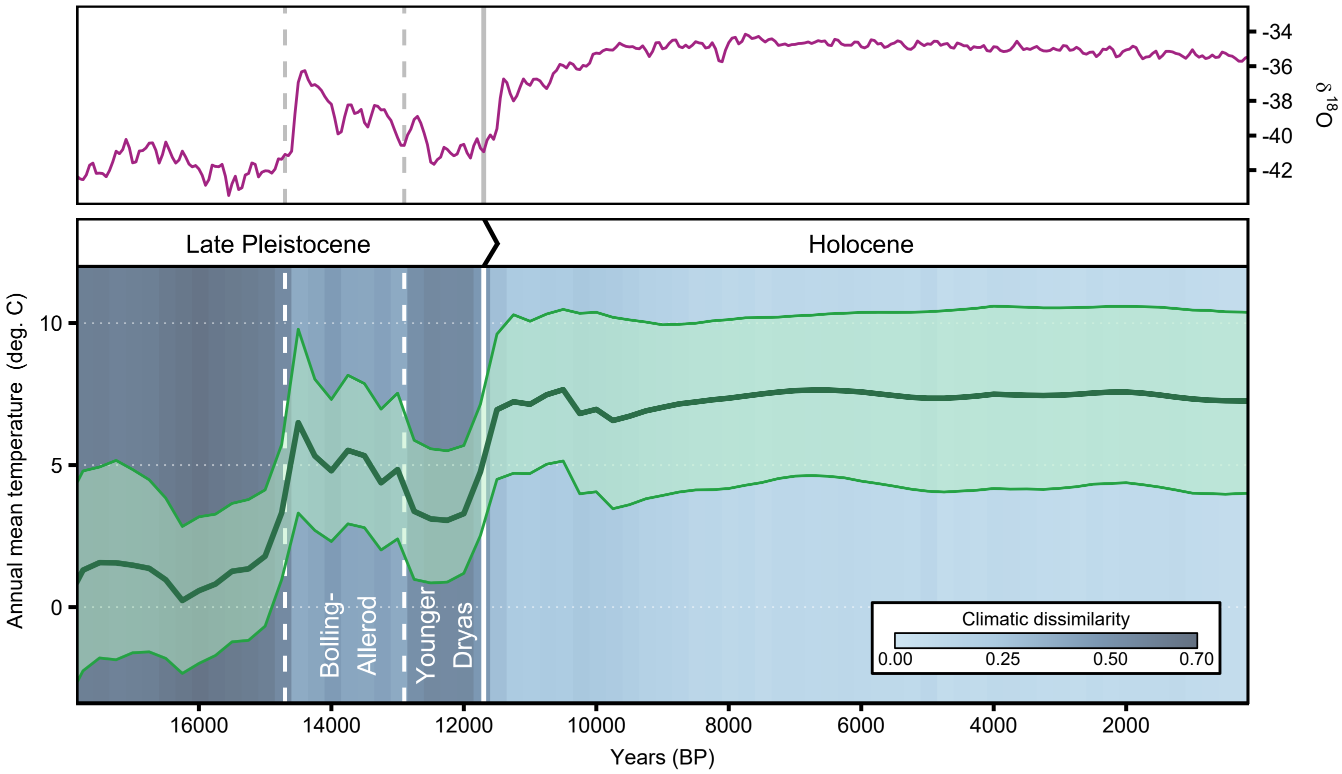

Fig. S4. Chronology of past climate across the Late Pleistocene and the Holocene. Upper panel shows the evolution of North Greenland Ice-Core Project (NGRIP) oxygen isotope 18 values (permille) as 50 year mean values [13]. Lower panel shows the average annual temperature across Europe, from HadCM3B simulations [14]. Shaded area represents interquartile range. Blue background represents the level of climatic dissimilarity (see Methods).

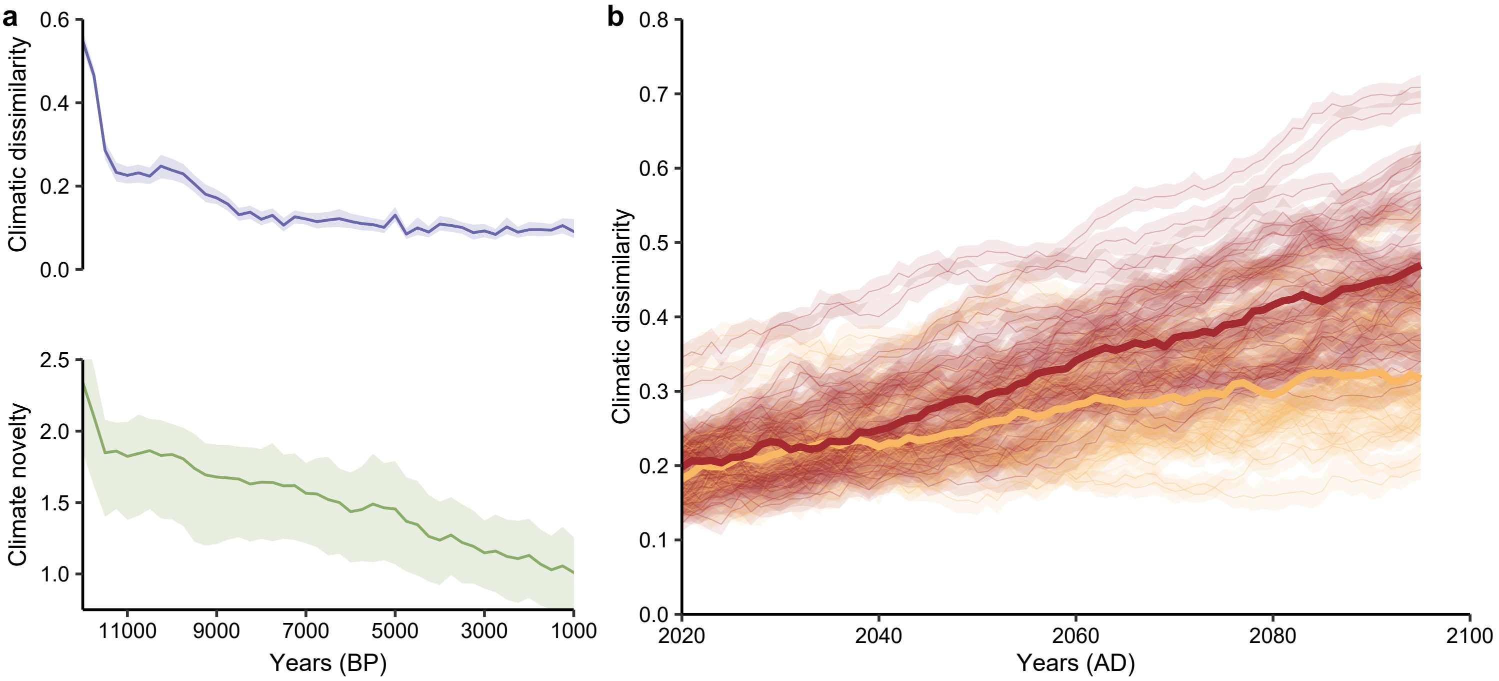

Fig. S5. Climate dissimilarity. (a) Climatic dissimilarity as calculated in this paper and climate novelty calculated following [15]. The reference period is 1901-2000. **(b)** Evolution of future climatic dissimilarity, across the 34 GCM simulations [16]. Yellow and red correspond to SSP245 and SSP585 scenarios. Shaded areas represent 95% confidence interval for climatic dissimilarity/interquartile range for climate novelty.

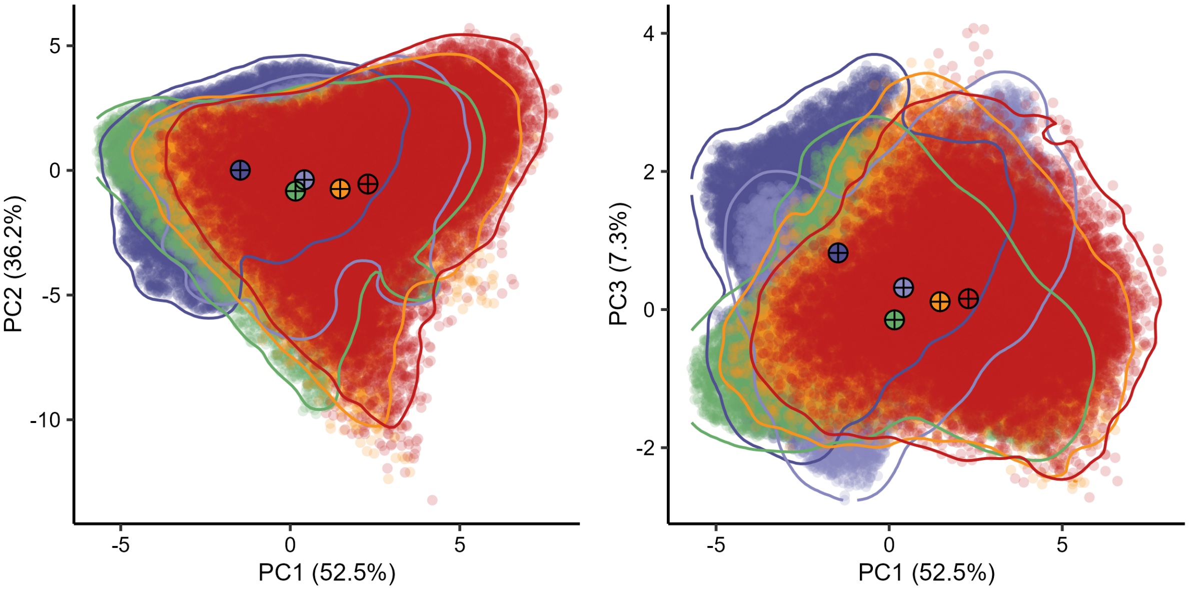

Fig. S6. Climate hypervolumes for Early Holocene period (dark and light blue, respectively 12000 BP and 11500 BP) and 2090 projections (orange and red, respectively SSP2 and SSP5 scenarios), as compared to historical period (green, 1901-2000). Hypervolume contours are generated using a two-dimensional kernel density estimation. Bigger points represent hypervolume centroids. Note that SSP2 and SSP5 points where randomly sampled in the 34 GCM hypervolumes used in this study [16]. PC1-3 correspond to the first three principal component axis from three-month means temperature and three-month sums of precipitation (see Methods), with the percentage of variation explained in parenthesis. Temperature contributes mostly to PC1, whereas precipitation contributes mostly to PC2 and PC3.

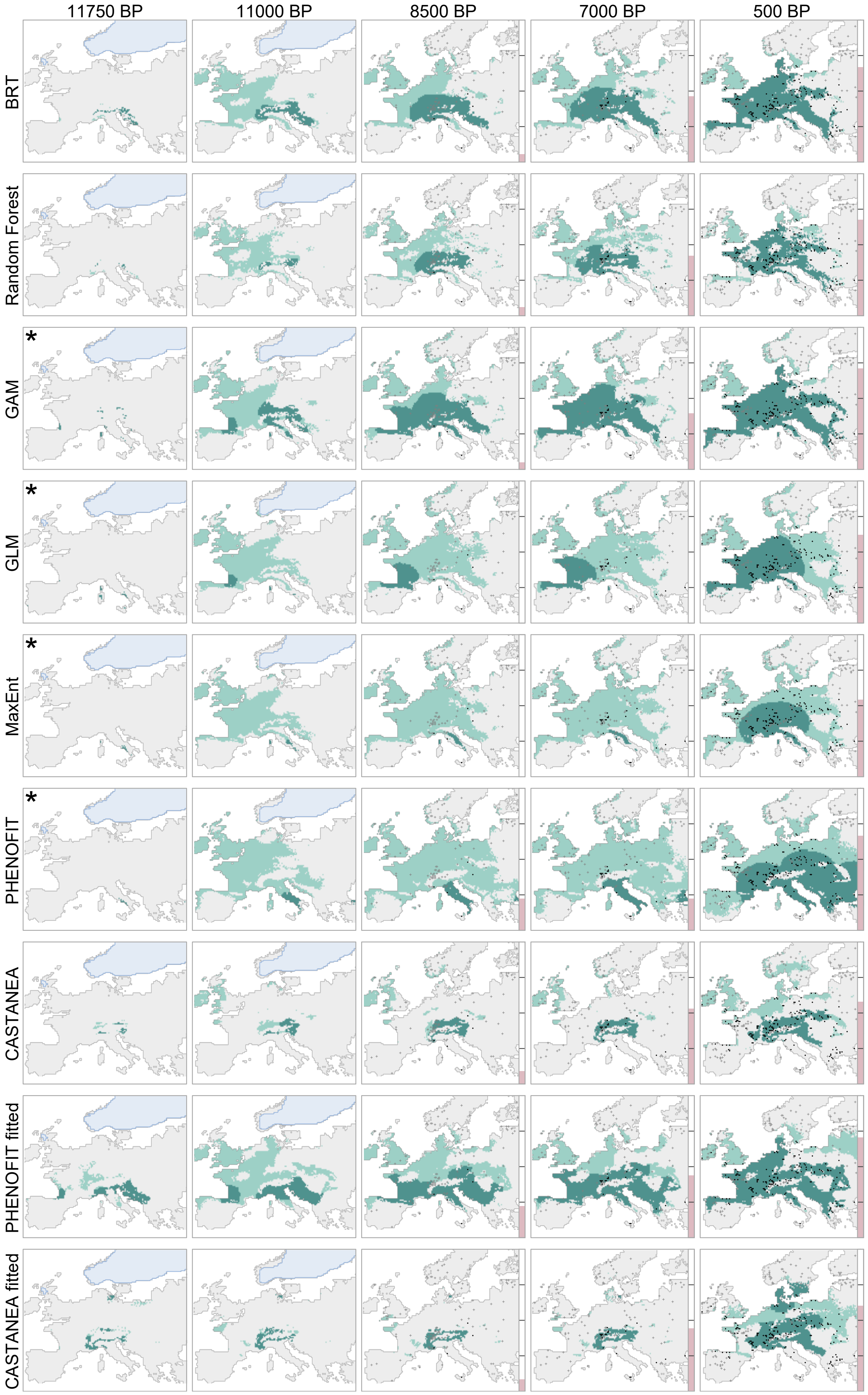

Fig. S7. Example of paleosimulations obtained with the nine models used in this study for beech (*Fagus sylvatica*). The five first rows correspond to the five correlative models (boosted regression tree, down-sampled random forest, generalized additive model, generalized linear model with lasso regularization, MaxEnt). The four last rows correspond to two different versions (expert calibration and inverse calibration using occurrence data) of two explicit-based models (PHENOFIT and CASTANEA). Light green area is the modelled suitable area, dark green area is the colonized area (after migration). Light blue represents the ice sheet extent. Black dots are deciduous oak fossil pollen presences (based on fossil pollen), grey crosses are absences. The vertical pink gauge represents model predictive performance (Sørensen index, $[0, 1]$, ticks every 0.25). The model for which migration started at 11.75 kyr BP rather than 12 kyr BP is marked with an asterisk. "BP" stands for "before present" (1950).

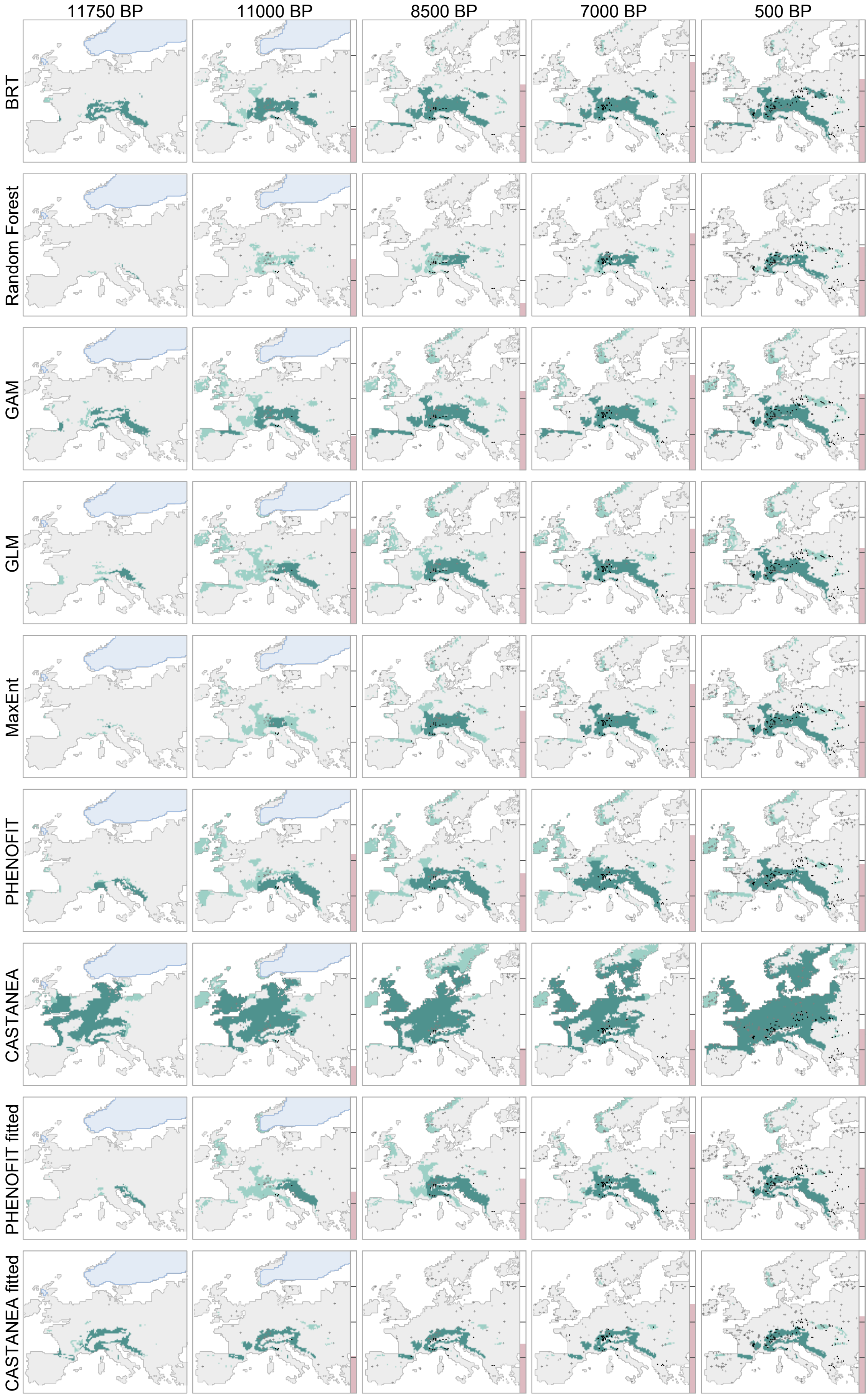

Fig. S8. Example of paleosimulations obtained with the nine models used in this study for fir (*Abies alba*). Same caption as Fig. S7.

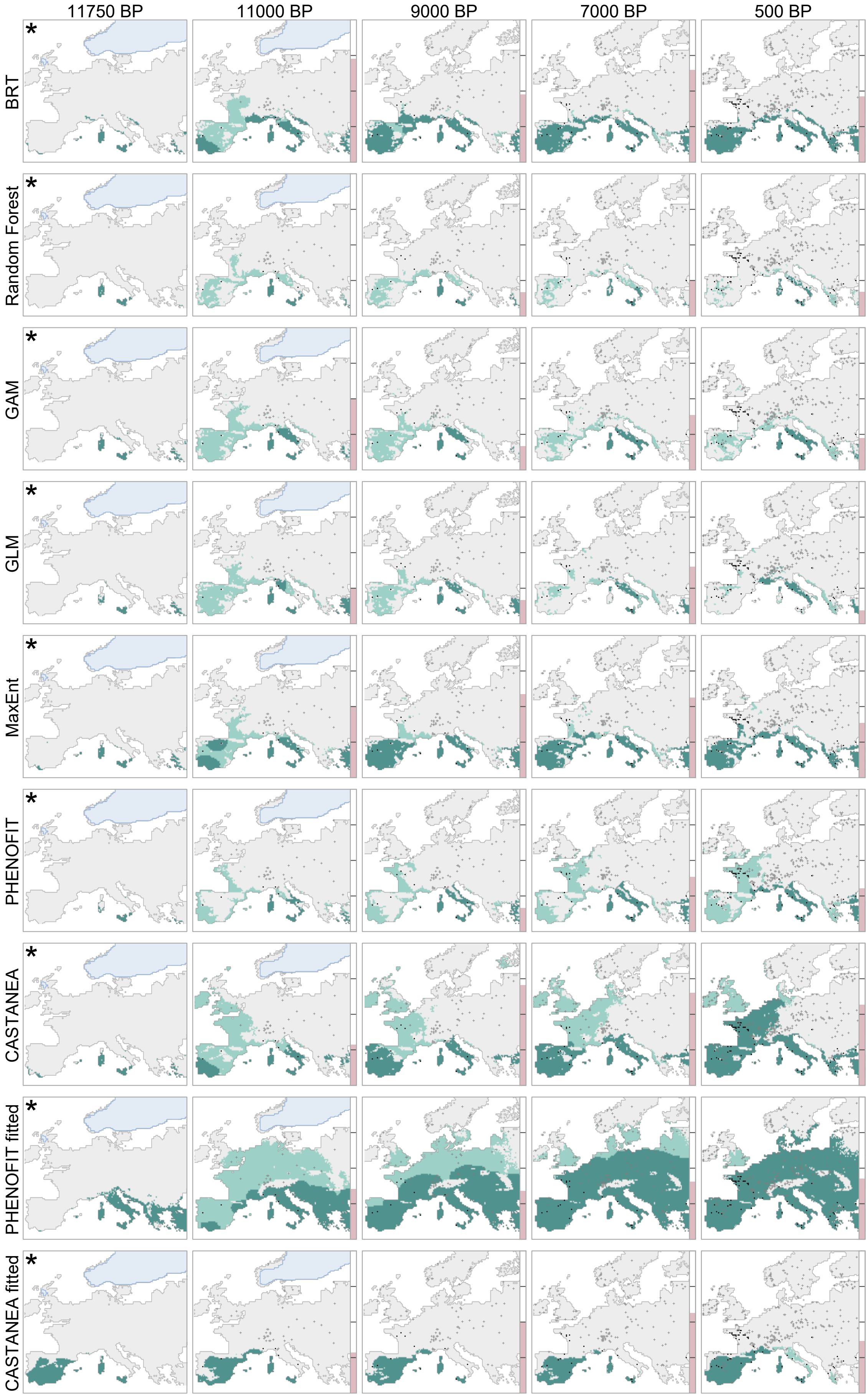

Fig. S9. Example of paleosimulations obtained with the nine models used in this study for evergreen oak (*Quercus ilex*). Same caption as Fig. S7.

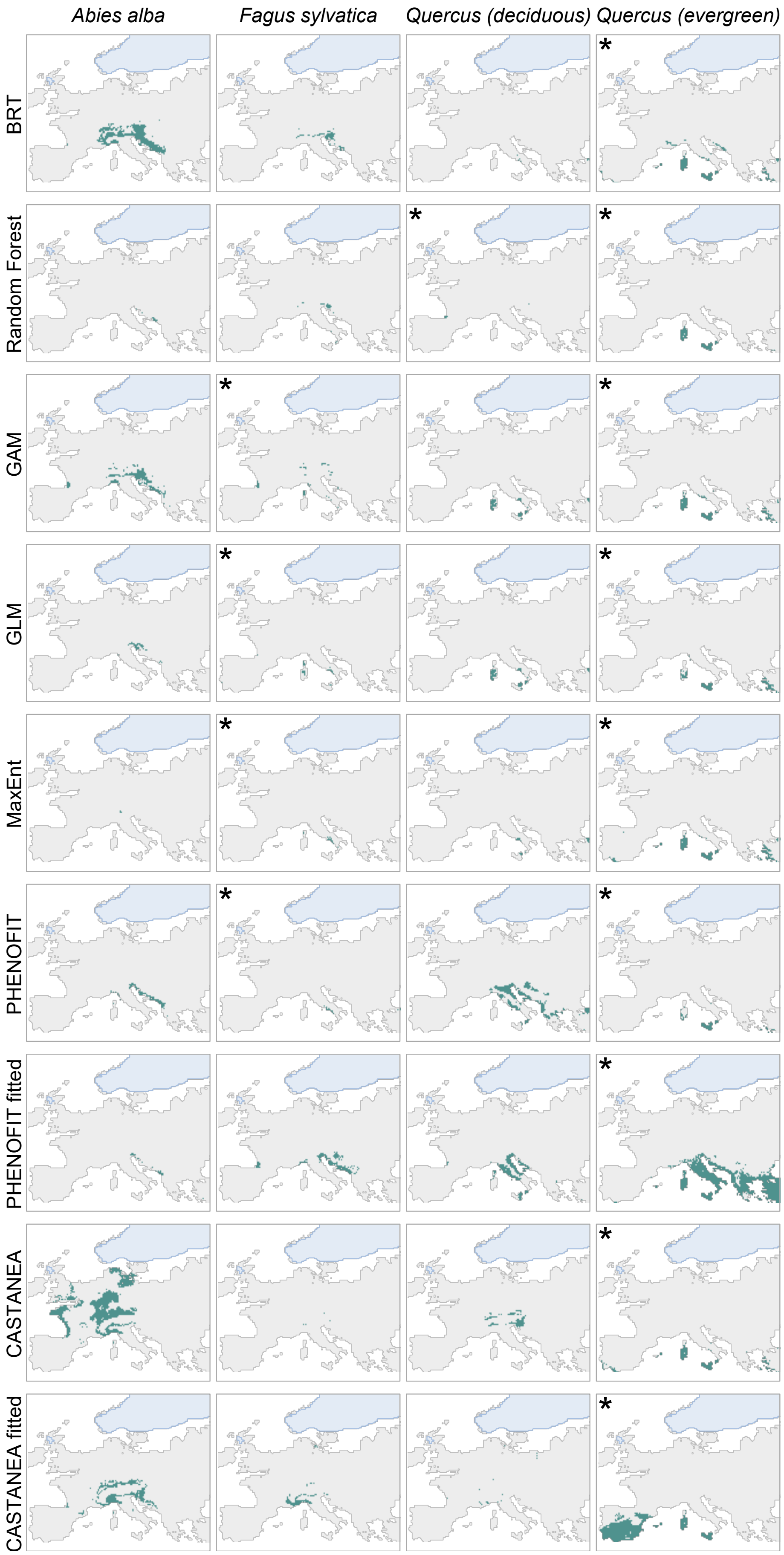

Fig. S10. Initial distributions predicted by the different models. These initial distributions are the starting points for the migration cellular automaton (see Methods in the main text). Models for which migration started at 11.75 kyr BP rather than 12 kyr BP are marked with an asterisk.

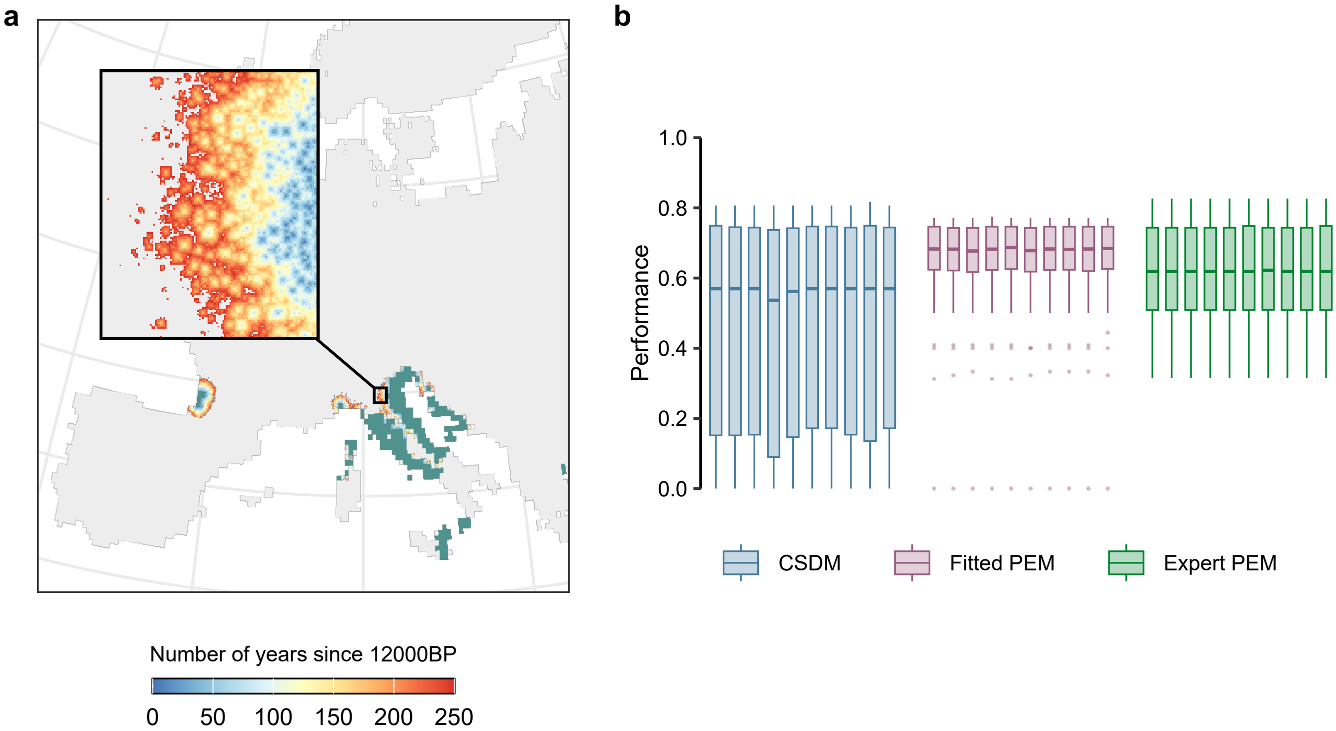

Fig. S11. (a) Illustration of the first 250 years of deciduous oak migration process, with PHENOFIT fitted model. Dark green area represents migration starting points at 12000 BP. **(b)** Variation of model performance for deciduous *Quercus*, due to the stochasticity of migration simulations.

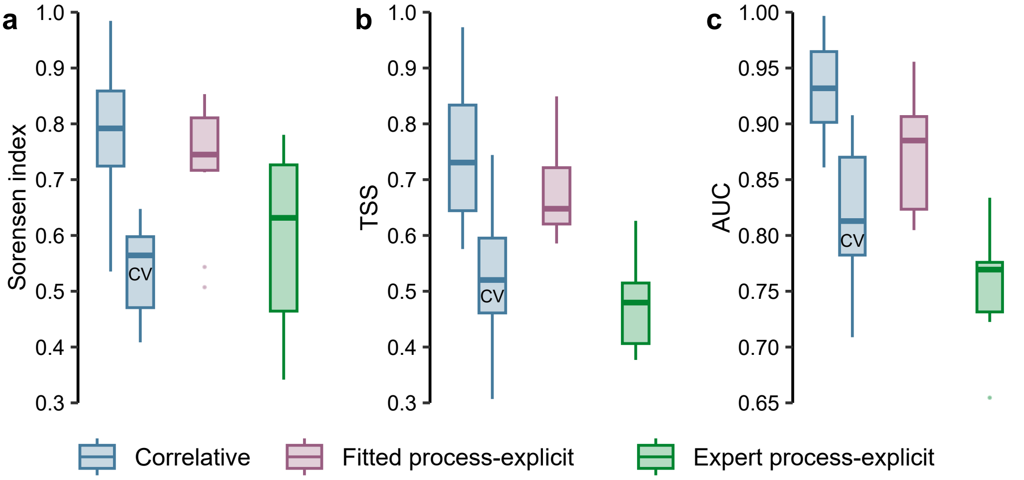

Fig. S12. Model performance in the 1970-2000 period, measured with three different metrics: (a) Sorensen index, (b) TSS and (c) AUC. "CV" stands for "cross-validation", when correlative extrapolation errors were assessed using a block cross-validation method.

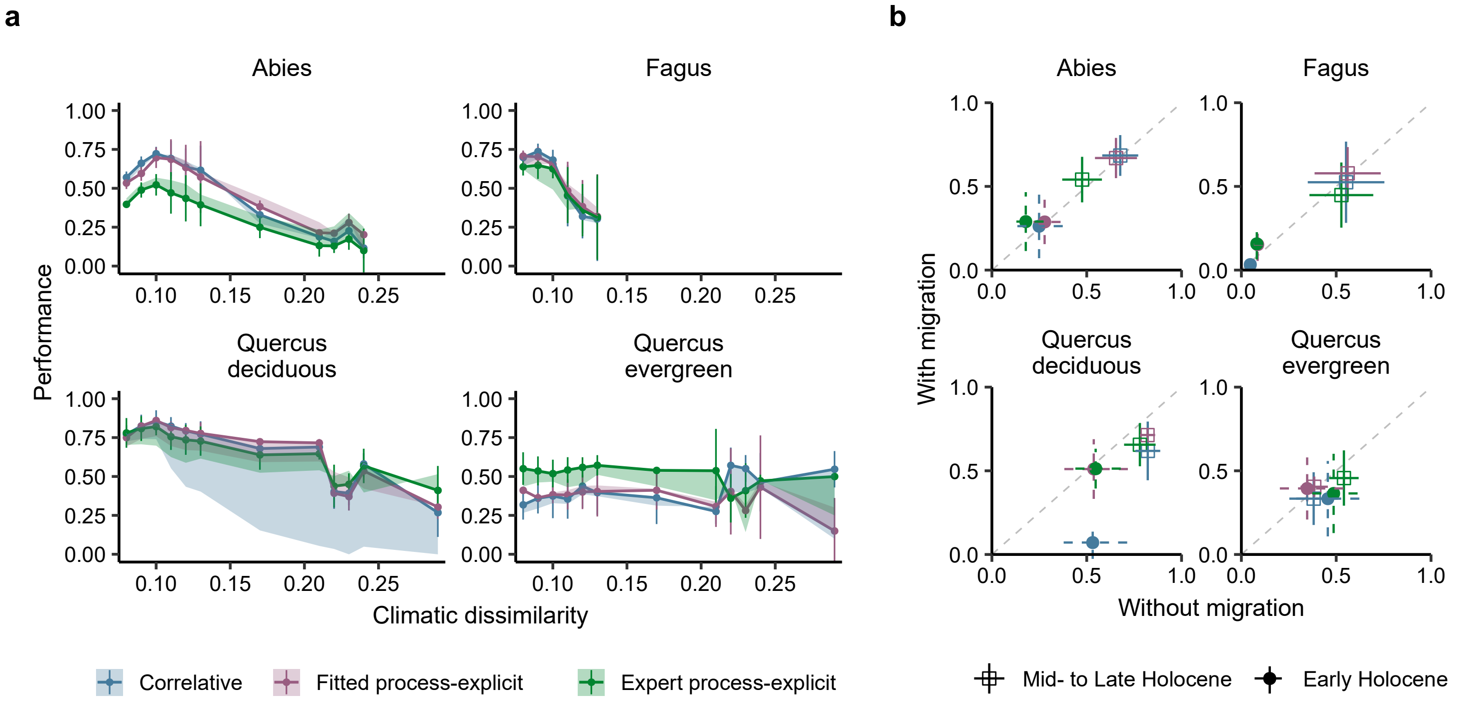

Fig. S13. Impacts of migration on model performance. (a) Evolution of model performance against climatic dissimilarity. Lines and points represent model performance without migration, shaded areas show performance evolution when simulating migration. Panel **(b)** displays model performance with and without migration, for the Early Holocene (> 8.2 kyr BP) and the Mid- to Late Holocene (< 8.2 kyr BP).

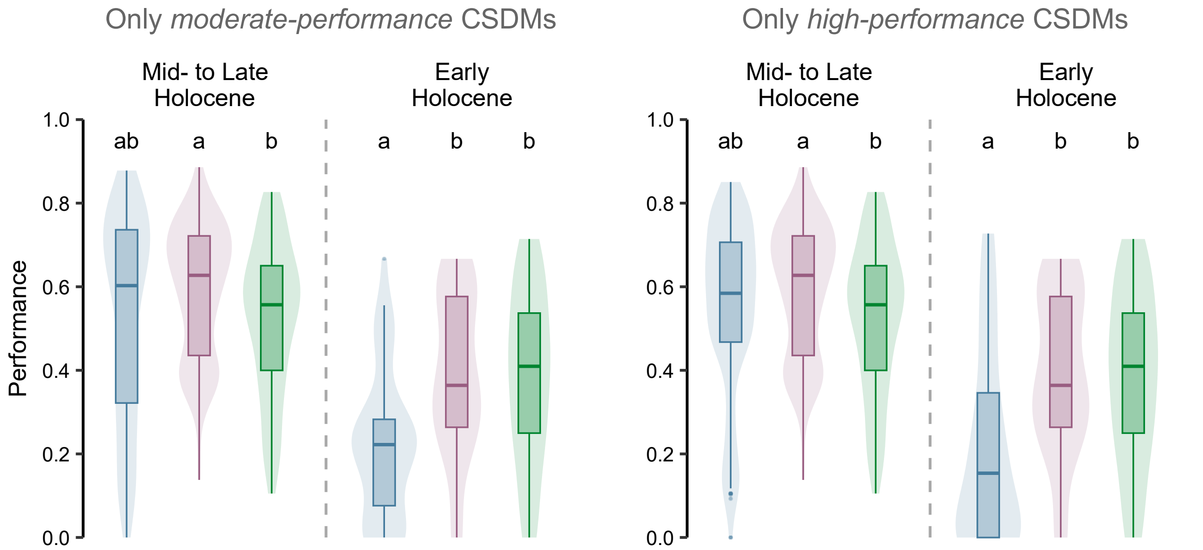

Fig. S14. Impacts of CSDM class on model performance, during the Early Holocene (> 8.2 kyr BP) and the Mid- to Late Holocene (< 8.2 kyr BP). Following [6], high-performance models correspond to Random Forest, BRT and MaxEnt, and moderate-performance models correspond to GLM and GAM. The grouping letters represent the multiple comparisons with pairwise Conover-Iman tests.

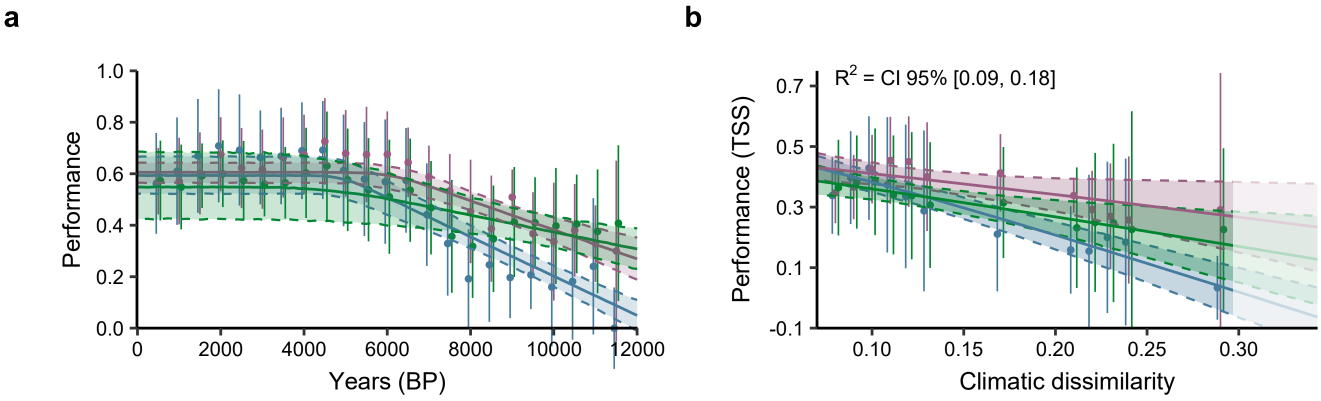

Fig. S15. (a) Model performances against time. Lines are linear-plateau regressions, which follow two phases (a flat plateau and a linear response). Shaded areas represents 2.5% and 97.5% confidence intervals, calculated with the R package *propagate* [17] by using first and second-order Taylor expansion and Monte Carlo simulations. **(b)** Bayesian beta regression of model performance (TSS) against climatic dissimilarity, with the same simulations as in main text (migration started from 12 kyr BP, or 11.75 kyr BP when a model simulates no presence at 12 kyr BP).

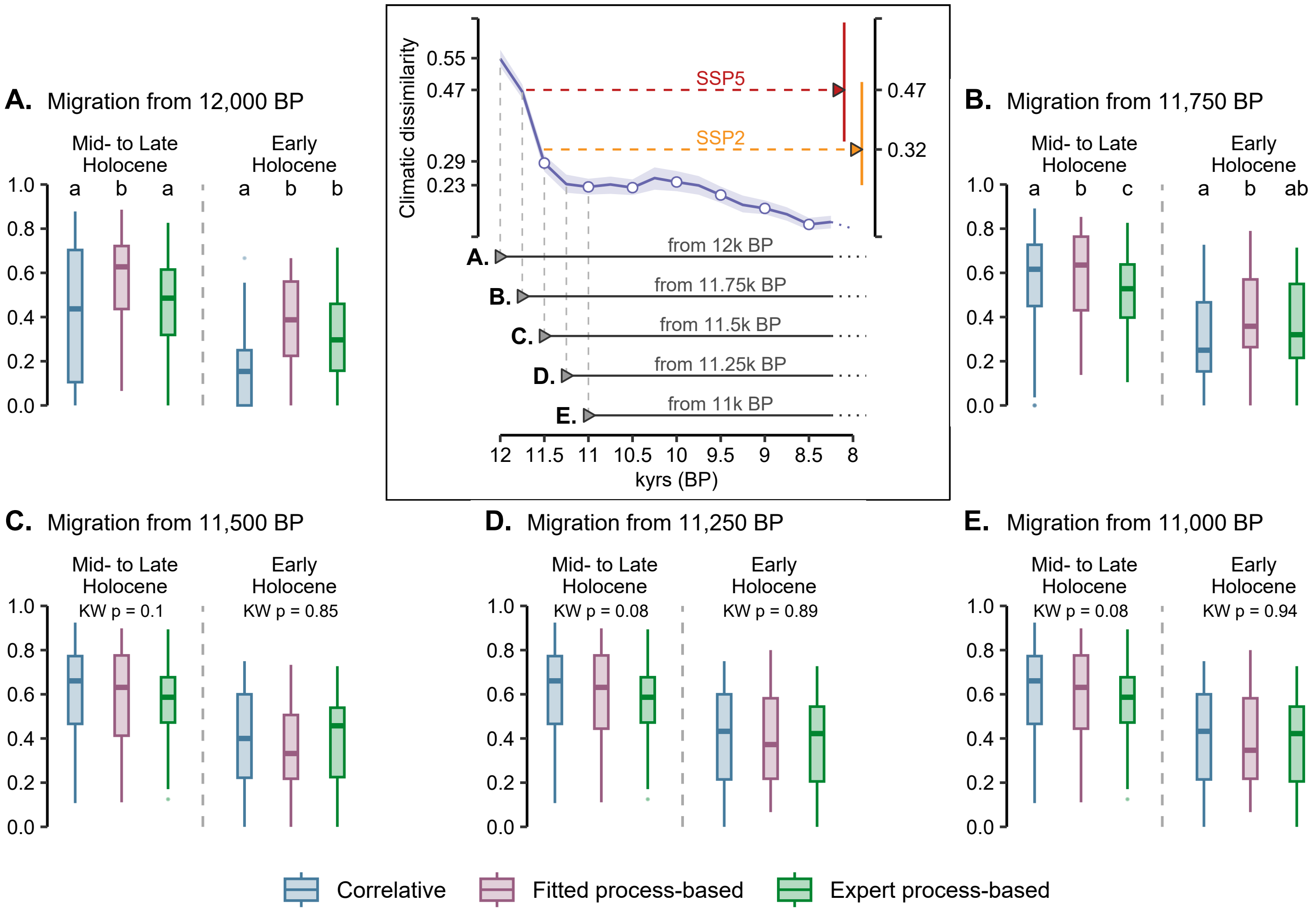

Fig. S16. Performance of correlative models, fitted process-explicit models (inverse calibration using occurrence data) and expert process-explicit models (classical calibration), with different initial periods for migration. (Upper middle plot) Different climatic dissimilarity breadths covered by the different simulations. Climatic dissimilarity is computed as 1-Sørensen similarity between bootstrapped climatic hypervolumes. Blue line represents median dissimilarity, shaded area represents 90% confidence intervals, based on HadCM3B model. Points on the blue line represent periods on which model projections are evaluated against fossil pollen records. Yellow and red triangles indicate the expected level of climatic dissimilarity by the end of the 21st century (2090-2100) for SSP2-4.5 and SSP5-8.5 scenarios, predicted by 34 global climate models of NEX-GDDP-CMIP6. Yellow and red vertical lines represent 90% confidence intervals. **(A,B,C,D,E)** Difference in performance (Sørensen index) across models, during the Early Holocene ($>$ 8.2 kyr BP) and the Mid- to Late Holocene ($<$ 8.2 kyr BP). "KW" stands for "Kruskal-Wallis". If the null hypothesis of the KW test is rejected, the grouping letters represent the multiple comparisons with pairwise Conover-Iman tests.

References

[1] Mauri, A., Strona, G. & San-Miguel-Ayanz, J. EU-Forest, a high-resolution tree occurrence dataset for Europe. *Scientific Data* **4**, 160123 (2017). URL https://www.nature.com/articles/sdata2016123.

[2] GBIF. The global biodiversity information facility (2022). URL https://www.gbif.org.

[3] Jalas, J. & Suominen, J. *Atlas Florae Europaeae* (Committee for Mapping the Flora of Europe and Societas Biologica Fennica Vanamo, Helsinki, Finland, 1972–2005).

[4] Bohn, U. *et al.* Map of the natural vegetation of europe - scale 1:2500000 (2003). URL https://www.synbiosys.alterra.nl/eurovegmap/.

[5] Monnet, A.-C. *et al.* WOODIV, a database of occurrences, functional traits, and phylogenetic data for all Euro-Mediterranean trees. *Scientific Data* **8**, 89 (2021). URL https://www.nature.com/articles/s41597-021-00873-3.

[6] Valavi, R., Guillera-Arroita, G., Lahoz-Monfort, J. J. & Elith, J. Predictive performance of presence-only species distribution models: a benchmark study with reproducible code. *Ecological Monographs* **92**, e01486 (2022). URL https://onlinelibrary.wiley.com/doi/abs/10.1002/ecm.1486.

[7] Van der Meersch, V. & Chuine, I. Estimating process-based model parameters from species distribution data using the evolutionary algorithm CMA-ES. *Methods in Ecology and Evolution* **14**, 1808–1820 (2023). URL https://onlinelibrary.wiley.com/doi/abs/10.1111/2041-210X.14119.

[8] Hansen, N. & Ostermeier, A. Completely Derandomized Self-Adaptation in Evolution Strategies. *Evolutionary Computation* **9**, 159–195 (2001).

[9] Saltré, F. *et al.* Climate or migration: what limited European beech post-glacial colonization? *Global Ecology and Biogeography* **22**, 1217–1227 (2013). URL https://onlinelibrary.wiley.com/doi/abs/10.1111/geb.12085.

[10] Duputié, A., Rutschmann, A., Ronce, O. & Chuine, I. Phenological plasticity will not help all species adapt to climate change. *Global Change Biology* **21**, 3062–3073 (2015). URL https://onlinelibrary.wiley.com/doi/abs/10.1111/gcb.12914.

[11] Gauzere, J. *et al.* Where is the optimum? Predicting the variation of selection along climatic gradients and the adaptive value of plasticity. A case study on tree phenology. *Evolution Letters* **4**, 109–123 (2020). URL https://onlinelibrary.wiley.com/doi/abs/10.1002/evl3.160.

[12] Dufrêne, E. *et al.* Modelling carbon and water cycles in a beech forest: Part I: Model description and uncertainty analysis on modelled NEE. *Ecological Modelling* **185**, 407–436 (2005). URL https://www.sciencedirect.com/science/article/pii/S0304380005000098.

[13] Andersen, K. K. *et al.* High-resolution record of Northern Hemisphere climate extending into the last interglacial period. *Nature* **431**, 147–151 (2004). URL https://www.nature.com/articles/nature02805.

[14] Armstrong, E., Hopcroft, P. O. & Valdes, P. J. A simulated Northern Hemisphere terrestrial climate dataset for the past 60,000 years. *Scientific Data* **6**, 265 (2019). URL https://www.nature.com/articles/s41597-019-0277-1.

[15] Burke, K. D. *et al.* Differing climatic mechanisms control transient and accumulated vegetation novelty in Europe and eastern North America. *Philosophical Transactions of the Royal Society B: Biological Sciences* **374**, 20190218 (2019). URL https://royalsocietypublishing.org/doi/10.1098/rstb.2019.0218.

[16] Thrasher, B. *et al.* NASA Global Daily Downscaled Projections, CMIP6. *Scientific Data* **9**, 262 (2022). URL https://www.nature.com/articles/s41597-022-01393-4.

[17] Spiess, A.-N. *propagate: Propagation of Uncertainty* (2018). URL https://CRAN.R-project.org/package=propagate. R package version 1.0-6.
